# Multiomics integration-based immunological characterizations of adamantinomatous craniopharyngioma in relation to keratinization

**DOI:** 10.1038/s41419-024-06840-1

**Published:** 2024-06-21

**Authors:** Chunming Xu, Jie Wu, Jiye Ye, Yuancheng Si, Jinshi Zhang, Bowen Wu, Laisheng Pan, Jun Fu, Quan Ren, Shenhao Xie, Bin Tang, Yingqun Xiao, Tao Hong

**Affiliations:** 1 Jiangxi Key Laboratory of Neurological Diseases, Department of Neurosurgery, The First Affiliated Hospital, Jiangxi Medical College, Nanchang University, Nanchang, Jiangxi China; 2 Department of Mathematics, University of Manchester, Manchester, UK; 3 The School of Economics, Fudan University, Shanghai, China; 4 Department of Pathology, Affiliated Infectious Disease Hospital of Nanchang University, Nanchang, Jiangxi China

**Keywords:** CNS cancer, Tumour immunology

## Abstract

Although adamantinomatous craniopharyngioma (ACP) is a tumour with low histological malignancy, there are very few therapeutic options other than surgery. ACP has high histological complexity, and the unique features of the immunological microenvironment within ACP remain elusive. Further elucidation of the tumour microenvironment is particularly important to expand our knowledge of potential therapeutic targets. Here, we performed integrative analysis of 58,081 nuclei through single-nucleus RNA sequencing and spatial transcriptomics on ACP specimens to characterize the features and intercellular network within the microenvironment. The ACP environment is highly immunosuppressive with low levels of T-cell infiltration/cytotoxicity. Moreover, tumour-associated macrophages (TAMs), which originate from distinct sources, highly infiltrate the microenvironment. Using spatial transcriptomic data, we observed one kind of non-microglial derived TAM that highly expressed GPNMB close to the terminally differentiated epithelial cell characterized by RHCG, and this colocalization was verified by asmFISH. We also found the positive correlation of infiltration between these two cell types in datasets with larger cohort. According to intercellular communication analysis, we report a regulatory network that could facilitate the keratinization of RHCG^+^ epithelial cells, eventually causing tumour progression. Our findings provide a comprehensive analysis of the ACP immune microenvironment and reveal a potential therapeutic strategy base on interfering with these two types of cells.

## Introduction

Adamantinomatous craniopharyngiomas (ACPs), one kind of epithelial tumour that develops from remnants of Rathke’s pouch, are relatively rare in adults [1]. ACPs are histologically heterogeneous and characterized by cysts, calcifications and other solid components [2]. One typical pathological feature of ACP is the accumulation of keratins (known as ghost cells), meanwhile pathological staining reveals other features, including palisading epithelium (PE), stellate reticulum (SR) and whorl-like cell clusters (WC) with nucleo-cytoplasmic β-catenin accumulation [3]. The primary function of keratins is structural support, and they are considered to play an inhibitory role in migration [4]. However, the abnormal build-up of keratins in ACPs will inevitably lead to tumour enlargement, as they occupy at least one-third of the tumours [5]. In particular, agglomerated keratins have been considered critical structures related to calcification; these pathological structures greatly increase the difficulty of surgery [6]. To date, the molecular mechanisms that drive the formation of these specific pathological structures are poorly understood.

For ACP, there are very few therapeutic options other than surgery. And because ACPs usually occur in the sellar/suprasellar region, near the optic chiasma, and often involve the pituitary stalk and the hypothalamus [7], gross-total resections become rather challenging. Subtotal resections with irradiation are often accompanied by tumour growth or irradiation-related side effects [2]. Hence, patients could experience a wide range of complications caused by tumour progression and therapeutic interventions, including panhypopituitarism, hypothalamic dysfunction, inhibited growth and sexual maturation in children, altogether leading to poor prognosis [8, 9]. It is necessary to understand the mechanism of cellular and molecular remodelling in the tumour microenvironment (TME) of ACP to find potential intervention targets.

Cell‒cell interactions between different cell types in the TME are critical for tumour progression, metastasis, and therapeutic responses [10]. For the moment, the identity of different cells in ACP is still not fully explored. Previous studies have used mRNA expression microarray or bulk RNA-seq combined with laser capture microdissection to explore the transcriptomic alterations in ACP [11–13]. However, these strategies do not have the power to detect cell-type-specific changes. Currently, no studies have been performed to investigate the composition of the immunological microenvironment in ACP by using single-nucleus RNA sequencing (snRNA-seq) and spatial transcriptomics (ST). In this manuscript, we investigated the composition of the TME in ACP by snRNA-seq and ST assays and explored the molecular characteristics and potential functions of the different cell classifications. Our research identified that GPNMB^+^ TAMs and RHCG^+^ epithelial cells are enriched in keratin regions, which suggests that the complex interplay between these two cells may play an important role in the formation of keratin and could serve as potential targets for ACP therapy.

## Results

### snRNA-seq landscape of ACP reveals high immune cell infiltration in the TME

**Fig. 1:**
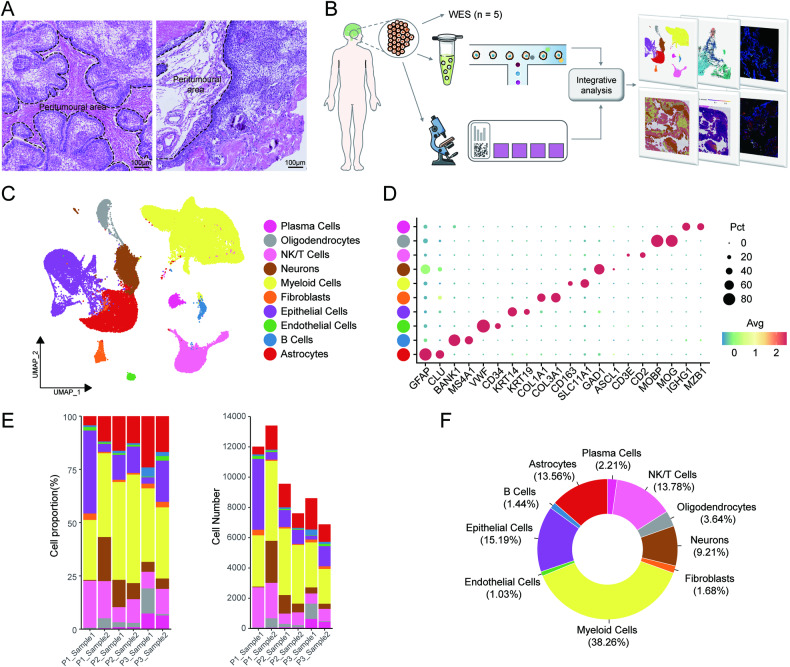
The cellular composition and proportions in ACP as determined by snRNA-seq. **A** Representative histology of peritumoural areas near (left; replete with glial tissue) and far from hypothalamus (right; adjacent to loose fibrous stroma with blood vessels) in ACP. Scale bar: 100 μm. **B** Scheme of the overall study procedure. The ACP tissues adjacent to the hypothalamus and paired ACP tissues away from the hypothalamus were collected for snRNA-seq and spatial transcriptomic analysis. **C** UMAP of 58,081 nuclei showing 10 clusters in different colours. **D** Dot plot showing marker genes for 10 clusters coloured by expression levels. The dot size represents the proportion of nuclei that expressed the genes in each cluster. **E** Stacked bar plots showing proportions and cell numbers of 10 clusters in each sample, and (**F**) pie chart indicating the overall percentage of each cluster in ACP.

Considering peritumoural tissues showed differences in composition based on where it located (Fig. 1A), we respectively collected tumour tissues adjacent to (named sample 1) and away from the hypothalamus (named sample 2) from 3 ACP patients for snRNA-seq to provide a more holistic assessment of TME and tumour heterogeneity (Fig. 1B; Table S1). Exome sequencing revealed the mutation pattern of CTNNB1 in our cohort. After quality control and filtering of the data, a total of 58,081 nuclei and 29,064 genes were retained. We identified 10 major cell types according to the expression of their shared canonical markers (Fig. 1C), including astrocytes (*n* = 7875), B cells (*n* = 839), endothelial cells (*n* = 600), epithelial cells (*n* = 8825), fibroblasts (*n* = 976), myeloid cells (*n* = 22219), neurons (*n* = 5351), NK/T cells (*n* = 8002), oligodendrocytes (*n* = 2112) and plasma cells (*n* = 1282) (Fig. 1D). We quantified the changes in cellular composition among specimens and found the samples away from the hypothalamus still contained neurons (Fig. 1E), suggesting that the distal specimens may not be as distant as we expected. However, this did not materially affect our exploration of the intratumoural heterogeneity. Furthermore, among all cell types, the number of myeloid cells (38.26%) was the largest, and the NK/T cell proportion (13.78%) was not low, which indicated that there was abundant immune cell infiltration in the TME (Fig. 1F).

### NK/T-cell phenotypes suggest an immunosuppressive status in the ACP microenvironment

**Fig. 2:**
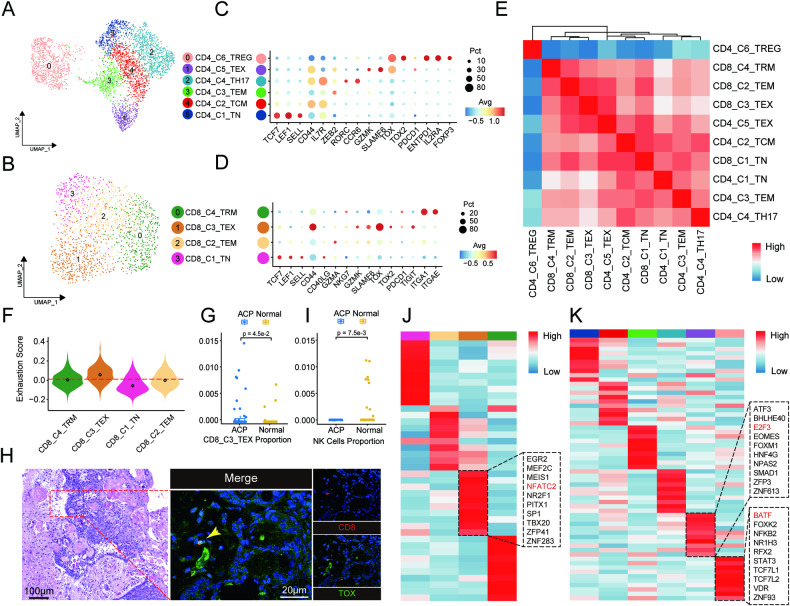
Immunological features of T cells in ACP. **A**, **B** UMAP of CD4^+^ and CD8^+^ T cells, showing 6 and 4 subclusters in different colours, respectively. **C**, **D** Dot plots of marker genes for CD4^+^ T cells and CD8^+^ T cells coloured by expression levels. **E** Heatmap showing the combined correlation between the inferred cell types of CD4^+^ and CD8^+^ T cells. **F** Violin plot showing the exhaustion score in CD8^+^ T cells. The dashed line indicates the median signature score, and the rhombus point in each violin represents its own median score. **G** Comparison of the infiltration proportion of CD8_C3_TEX between ACP samples and normal brain samples in the GSE94349 and GSE68015 datasets. Boxes show the median ± 1 quartile, and the p value is annotated. **H** Representative staining of Tex cells in ACP. The yellow arrows indicate CD8^+^ Tex cell. CD8 (red) and TOX (green) are shown in individual channels with DAPI (blue). Scale bar: 100 μm (HE) and 20 μm (mIHC). **I** Comparison of NK cell infiltration proportions between ACP samples and normal brain samples. **J**, **K** Heatmaps showing the normalized TF activity of the top 10 regulons for each subcluster of CD8^+^ T cells and CD4^+^ T cells. Subclusters are coloured as in UMAP. The dotted black boxes indicate the top 10 regulons in specified subcluster, only the regulons of interest are labelled in red.

To understand the extent of lymphocyte infiltration in ACP, we decided to scrutinize the NK/T-cell landscape of tumours. Unbiased clustering was used to initially identify 6 major immune subpopulations, including CD4^+^ T cells marked by CD4, CD8^+^ T cells marked by CD8A and CD8B, NK cells marked by NCR1 and NCAM1, γδT cells with high expression of TRGC2 and TRG-AS1, mucosal-associated invariant T cells (MAITs) positive for RORA and SLC4A10, and TCR^+^ macrophages characterized by CD4 and myeloid markers including CD163 and CD86 (Fig. S1A-B) [14–16]. CD4^+^ T cells and CD8^+^ T cells were further extracted and re-clustered into 6 clusters and 4 clusters respectively (Fig. 2A, B).

CD4_C1_TN/CD8_C1_TN clusters were characterized by naïve markers, such as TCF7, LEF1 and the homing receptor gene SELL (Fig. 2C, D). By contrast, the CD4_C2_TCM cluster was found to have higher expression of CD44 and IL7R, indicating the memory state (Fig. 2C, D). CD4_C3_TEM/CD8_C2_TEM clusters basically lost gene expression of naïve markers but expressed genes associated with effector memory cells, such as GZMA, GZMK, CD40LG (Fig. 2C, D) [15, 17]. The CD4_C4_TH17 cluster was positive for RORC and CCR6, which are considered Th17 cell markers (Fig. 2C, D) [15]. We also identified CD4_C6_TREG as the regulatory T (Treg) cell subtype by IL2RA, FOXP3 and the immunosuppressive gene ENTPD1 (Fig. 2C, D). The CD8_C4_TRM cluster was characterized by ITGA1 and ITGAE, which are recognized as classical tissue resident marker genes (Fig. 2C, D) [18].

The CD8_C3_TEX cluster was recognized as an exhausted T (Tex) cell subtype despite the missing expression of TOX2 and immune checkpoint genes. In comparison, we found this cluster highly expressed thymocyte selection-associated HMG box protein (TOX) and SLAMF6 which were all belonged to markers of T-cell exhaustion progenitors, suggesting a CD8^+^ Tex cell subtype with an early exhausted state (Fig. 2C, D) [19]. Surprisingly, we found that the CD4_C5_TEX cluster was also positive for the mentioned genes (Fig. 2C, D). Through a similar analysis based on the gene expression level, the result showed that the two TOX^+^ clusters tended to cluster together, which revealed a highly correlated expression pattern between CD8_C3_TEX and CD4_C5_TEX (Fig. 2E). Furthermore, we evaluated the extent of cell exhaustion for all subclusters of CD4^+^ T cells and CD8^+^ T cells by examining exhaustion signature genes [20]. The CD8_C3_TEX cluster was observed to have the highest exhaustion score among CD8^+^ T cells (Fig. 2F). For CD4_C5_TEX, this cluster also presented the highest exhaustion score except for the CD4_C6_TREG cluster, substantiating the existence of these two Tex subtypes (Fig. S1C). CIBERSORTx digital cytometry was performed to examine these T-cell subtypes across larger cohorts. We found that CD8^+^ Tex cells were significantly more abundant in ACP samples than in normal brain samples, demonstrating the enrichment of CD8^+^ Tex cells in the ACP TME (Fig. 2G). mIHC staining also confirmed the existence of CD8^+^ Tex cells (Fig. 2H). In addition, we evaluated the effector score among all CD4^+^ T-cell subsets and observed the highest score of Treg cells (Fig. S1D) [21]. Corresponding to that, TME of ACP was found to have low infiltration of high cytotoxic NK cells (Fig. 2I and S1E). All these results suggested an immunosuppressive TME in ACP.

To investigate the underlying regulation that drives the formation of the immunosuppressive TME, we evaluated the top 10 transcription factors (TFs) that are differentially activated in each subcluster by pySCENIC. We found that NFATC2, a pivotal regulator in Tex cells, was highly activated in the CD8_C3_TEX cluster and showed higher expression than other CD8^+^ subclusters (Fig. 2J and S1F) [22]. E2F3 was identified as a candidate critical TF in the CD4_C5_TEX cluster for the same reason. BATF, which can program Treg cells, had the highest transcriptional activity and a relatively high expression level in our data (Fig. 2K and S1G) [23]. In short, our data indicated an immunosuppressive TME accompanied by the generally low cytotoxicity of T cells in ACP.

### TAM subclusters show distinct functional phenotypes and variable sources

**Fig. 3:**
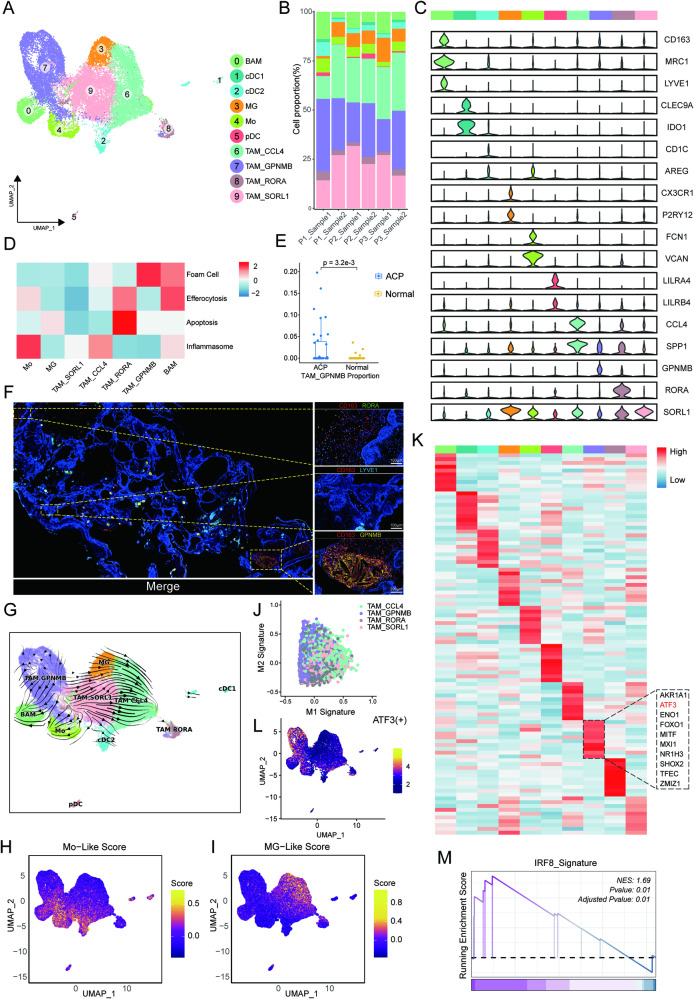
Characterization of myeloid cells in ACP. **A** UMAP of myeloid cells, with 10 subclusters in different colours. **B** Stacked bar plot showing the proportion of 10 subclusters in each sample. **C** Violin plot showing the expression of marker genes. **D** Differences in specific function-related phenotype activities scored by GSVA among subclusters of myeloid cells except 3 DC subclusters. The scores are z score normalized. **E** Comparison of the TAM_GPNMB infiltration proportion between ACP samples and normal brain samples. **F** Representative mIHC staining of CD163 (red), RORA (green), LYVE1(blue) and GPNMB (yellow) in ACP. 3 myeloid subclusters are shown in individual channels respectively on the right. Scale bar (right): 100 μm (top, middle) and 200 μm (bottom). **G** RNA velocity plot of myeloid cells showing the dynamic shift in the cell state. **H**, **I** UMAP plots showing the distribution of Mo-like/MG-like scores. **J** Scatterplot of M1 and M2 signature scores for each nucleus among 4 TAM subclusters. **K** Heatmap showing the normalized TF activity of the top 10 regulons for each subcluster in myeloid cells. The dotted black box indicates the top 10 regulons in TAM_GPNMB, and the regulon of interest is marked in red. **L** UMAP plot showing the expression of ATF3. **M** Enriched signature of IRF8 and its downstream gene signature in ACP by GSEA. NES, normalized enrichment score.

Myeloid cells, which make great contributions to shaping the TME, are highly heterogeneous cells [24]. By performing unsupervised graph-based clustering, we identified 10 myeloid subpopulations in all samples: 3 dendritic cell (DC) subclusters (cDC1, cDC2 and pDC) [25–27], 1 monocyte (Mo) subcluster [27], 2 brain-resident immune cell subclusters including microglia (MG) and border-associated macrophage (BAM) [28, 29], and 4 TAM subclusters. (Fig. 3A–C).

For TAMs, TAM_SORL1 cluster, which could mediate inflammatory responses, was similar to MG but without homeostatic genes, characterized by high expression of antigen-presenting genes (Fig. 3C and S2A, B). TAM_CCL4 was characterized by the expression of brain inflammation-associated chemokines, including CCL3L1, CCL4 and CCL4L2, similar to the activated MG reported in the brain (Table S2) [30, 31]. In comparison to other TAM subclusters, TAM_CCL4 was enriched in the positive regulation of immune response, suggesting the participation in immune activation (Fig. S2B). Meanwhile, consistent with the enrichment of VEGFA-VEGFR2 signalling pathway (Fig. S2B), we noticed a higher expression level of SPP1 in TAM_CCL4, which has been reported as a marker of angiogenesis-associated TAMs [32]. In view of the existence of inflammasomes in ACP, we tried to pinpoint the link association between inflammasomes and this TAM subcluster which had a strong ability with immune activation. As expected, the inflammasome scores were significantly elevated in TAM_CCL4 cluster (Fig. 3D). All results indicated that TAM_CCL4 has the dual functions of promoting tumour growth and mediating immune activity in ACP.

The TAM_GPNMB cluster, showing high activated GPNMB, constituted a large subgroup of myeloid cells. TAM_GPNMB represented one kind of tumour-specific macrophage, and the infiltration degree of TAM_GPNMB in ACP which was estimated by CIBERSORTx was significantly increased compared with that in brain (Fig. 3E). This subcluster expressed high levels of genes related to lipid metabolism, such as CD9, LIPA and LPL, similar to lipid-associated macrophages (Fig. S2A) [33–35]. We further quantified metabolic pathway activity by scMetabolism and found that multiple lipid metabolism-associated pathways were significantly enriched in the TAM_GPNMB cluster, including metabolism of lipids, triglyceride metabolism, fatty acid metabolism, sphingolipid metabolism and so on (Fig. S2C) [36]. This result highlighted that the TAM_GPNMB cluster was closely related to lipid metabolic reprogramming in the ACP TME. Meanwhile, differentially expressed gene (DEG) enrichment analysis revealed that the TAM_GPNMB cluster could promote epithelial cell migration had certain function of phagocytosis, which may be involved in the removal of dead tumour cells, suggesting a pivotal role for this cluster in the occurrence and development of tumours (Fig. S2B). The TAM_RORA cluster was identified by high RORA expression. Despite the low expression level, this TAM cluster also had upregulated lipid metabolism genes (Fig. S2A). We found that TAM_RORA had a high enrichment of the gene signatures for promoting apoptosis compared with other TAM clusters, indicating an apoptotic state (Fig. 3D). The intimate association of the TAM_GPNMB cluster with lipid metabolism reminded us of foam cells in ACP [37]. By examining related signature genes, we observed distinct functional statuses for these myeloid subclusters. Unsurprisingly, TAM_GPNMB had the highest foam cell gene signature score, closely followed by the BAM cluster [38]. Both TAM_RORA and BAM clusters were enriched in the efferocytosis pathway (Fig. 3D). We further confirmed by mIHC staining the existence of these cell subtypes in ACP (Fig. 3F). Significantly, the overall gene expression pattern was similar to aortic resident macrophages and TREM2hi macrophages of atherosclerosis, suggesting the similarity in the function or origin of these macrophage subclusters [39].

To investigate the ontogenies of various TAM subclusters in ACP, we first performed similarity analysis based on gene expression (Fig. S2D). The results showed that TAM_GPNMB had a higher correlation to Mo and BAM, while TAM_CCL4/TAM_SORL1 clusters had a similar expression pattern to MG. This illustrated the potential for diversity in TAM origin within ACP. Using RNA velocity, we also observed directional streams from MG/TAM_SORL1 clusters to the TAM_CCL4 cluster (Fig. 3G). Unexpectedly, RNA velocity showed a directional stream from the TAM_GPNMB cluster to BAM/Mo clusters (Fig. 3G). Based on the expression of MG-specific genes and Mo-specific genes collected from the literature, each nucleus in all subclusters was given scores [29]. The scoring results further supported the distinct ontogenies of TAM clusters, similar to what we described above (Fig. 3H, I). However, to fully elucidate the cell differentiation trajectory of TAMs in ACP, more lineage studies are needed.

Moreover, the scoring of M1 and M2 macrophage signatures was assessed in TAM clusters, and we found that all these subclusters generally co-expressed features of both M1 and M2 macrophages, suggesting that the simple polarization model was also not suitable for evaluating the state of TAMs in ACP (Fig. 3J) [27, 40]. Significantly, we observed that the TAM_GPNMB cluster tended to exhibit M2-polarized characteristics, which indicated a stronger ability for tumour promotion (Fig. S2E). This may be related to the different origins of each TAM cluster. Compared to MG-derived TAMs, Mo-derived TAMs have been reported to upregulate immunosuppressive cytokines and markers of lipid metabolism while preferentially aggregating in necrotic regions and exhibiting stronger phagocytosis [41]. Next, to further explore the regulatory network in different TAM subclusters, we performed pySCENIC to identify the particular TFs. Activating transcription factor 3 (ATF3), a key TF of innate immune response genes that can mediate anti-inflammatory activities in macrophages, was highly activated in the TAM_GPNMB cluster, further corroborating our above scorings (Fig. 3K, L) [42, 43].

Although the potential immunosuppressive mechanisms remain to be clarified, it has been reported that TAMs can promote T-cell exhaustion [44]. Given the characteristics of the ACP TME and abundant TAM infiltration, we tried to unearth the possible link between these two. GSEA showed an upregulation of IRF8 and its downstream gene signature in ACP tissues relative to normal brain tissues (Fig. 3M). IRF8 used to be considered a transcription factor associated with cDC1 differentiation and was recently regarded as a key TF in TAMs for presenting tumour cell antigens and driving T-cell exhaustion [45]. Similar to the literature, we observed that scores of IRF8 gene signature were generally flat or higher in each TAM subcluster compared with the cDC1 cluster, especially the TAM_GPNMB cluster, suggesting its important role in shaping the specific ACP TME (Fig. S2F). In summary, our extensive analysis results indicated that TAMs in ACP have different origins and diverse functions, and the TAM_GPNMB cluster may have a critical role in tumour progression.

### Tumour cells in ACP exhibit extensive transcriptional heterogeneity

**Fig. 4:**
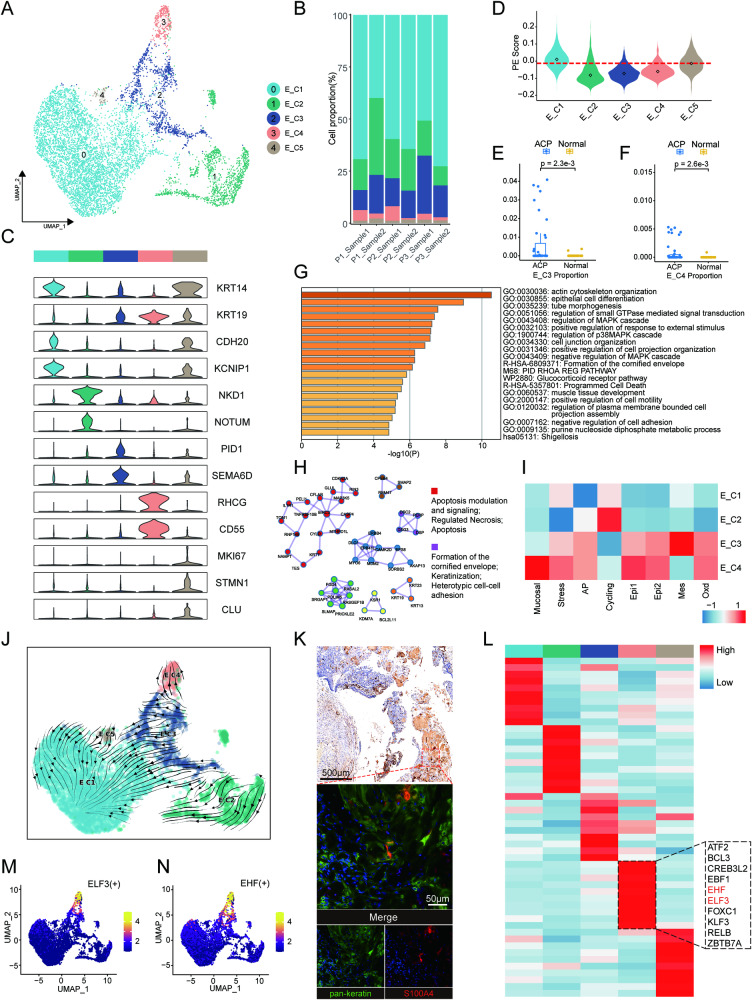
Characterization of epithelial cells in ACP. **A** UMAP of epithelial cells clustered into 5 subpopulations in different colours. **B** Stacked bar plot showing the proportion of 5 subclusters in each sample. **C** Violin plot showing the expression of marker genes. **D** Violin plot showing palisade epithelium score among epithelial subclusters. **E**, **F** Comparison of the infiltration proportion for the E_C3 and E_C4 clusters between ACP samples and normal brain samples. **G** Bar plot of DEG enriched pathways for the E_C4 cluster by the Metascape tool. **H** The PPI network showing 7 complexes automatically identified by the Molecular Complex Detection (MCODE) algorithm in Metascape, coloured by each identity. Two major functional labels are listed on the right. **I** Differences in 8 epithelial expression program activities scored by GSVA among subclusters of epithelial cells, except the E_C5 cluster, the proliferative subcluster. **J** RNA velocity plot of epithelial cells showing cell transition directions. **K** IHC staining showing positivity of S100A4. Scale bar: 500 μm. mIHC showing the colocalization between S100A4 (red) and pan-keratin (green) in KCs. Scale bar: 50 μm. **L** Heatmap showing the normalized TF activity of the top 10 regulons for each subcluster in epithelial cells. The dotted black box indicates the top 10 regulons in E_C4, and the regulon of interest is marked in red. **M**, **N** UMAP plots showing the expression of ELF3 and EHF.

ACPs have special histopathologic structures, but the functions and molecular characteristics of these heterogeneous structures remain elusive. By performing unsupervised clustering, we identified 5 distinct epithelial cell subclusters in all samples, and every subcluster had highly expressed marker genes and a specific gene expression pattern (Fig. 4A–C; Table S3). We utilized the muscat R package to assess the overall transcriptional similarity for all subclusters and the spectrum of expression differences reflected the reliability of our classification (Fig. S3A) [46]. Through asmFISH, we confirmed the presence of these epithelial subclusters (Fig. S3B, C).

For the E_C1 cluster, we noticed that most DEGs from E_C1 coincided with DEGs of PE obtained from laser capture microdissection performed by J. Apps et al. [11]. The scoring of the PE gene signature (top 100 genes) which was made based on DEGs reported by J. Apps et al. showed the apparently highest score in the E_C1 cluster, revealing its underlying PE identity in ACP (Fig. 4D). We continued to evaluate hallmark gene sets by GSVA, and it revealed a strong enrichment of cell cycle-related pathways in this subcluster. We also found it had a high proportion of S phase in the cell cycle, illustrating a strong proliferative ability (Fig. S3D–F). Assembly of collagen fibrils and integrin-mediated cell adhesion were identified as functional modules from the protein‒protein interaction (PPI) network in E_C1, hinting at its important role in the remodelling of the extracellular matrix at tumour fronts (Fig. S3G). The characteristic of the E_C2 cluster was the specific expression of target genes downstream of the Wnt signalling pathway (Fig. 4C), which indicated its identity of WC with nucleo-cytoplasmic β-catenin accumulation in ACP. The E_C2 cluster exhibited hyperactivation of the Wnt pathway and strong regulation of cell differentiation (Fig. S3D, E). PPI network analysis showed that its downstream effector proteins were related to the regulation of stem cell proliferation, indicating the stemness of WC (Fig. S3G). Notably, WC has been implicated as signalling hub in ACP [11], and we have previously found that there were different staining patterns in WC [47]. Thereby, E_C2 data was further extracted and identified into 3 subclusters through unsupervised clustering (Fig. S4A). All subclusters with a high and specific marker showed different secretion patterns of signalling factors (Fig. S4B–F). WC_1 was characterized by relatively high expression of FGF ligands (e.g. FGF3, FGF4, FGF12, FGF18, FGF19). WC_2 had high levels of BMP2 and BMP4. Moreover, SHH was highly expressed in this subcluster. WC_3 was identified by WNT ligands, including WNT6, WNT7A, WNT10A, WNT10B. These results revealed substantial heterogeneity inside WCs. GSVA also displayed different pathway activation patterns among these three subclusters, suggesting that different WC subclusters might play different roles in tumour development (Fig. S4G). The E_C3 cluster was considered as one kind of tumour-specific keratinocytes (KCs) (Fig. 4E). Odontogenesis and biomineral tissue development were enriched in this subcluster (Fig. S3E and S3G). By GSVA, we found that this subcluster was closely associated with the activation of multiple pathways, including angiogenesis, hypoxia and epithelial mesenchymal transition, revealed the role of the E_C3 cluster in tumour progression (Fig. S3D). The E_C4 cluster, which was also tumour-specific, was marked by RHCG (Fig. 4C, F). This cluster highly expressed CD55, a glycoprotein that can protect cells from complement-mediated attack, suggesting the clusters potential link with tumour immunity [48]. Immune-associated pathways from hallmark gene sets were also significantly enriched in the E_C4 cluster by GSVA, suggesting its potential effect in tumour immunity (Fig. S3D). In addition, DEG enrichment analysis and PPI network analysis revealed the participation of downstream proteins in apoptosis and keratinization ((Fig. 4G, H). We found that E_C4 was almost comprised of cells in the G1 phase of cell cycle, similar to senescent cells which undergone cell cycle arrest and marked by CDKN2A (Figs. S3F and S4H) [49]. CDKN2A was found to be expressed in WCs [50]. We therefore validated our analysis by IHC. Staining of RHCG and CDKN2A showed a similar location in the region of keratins (Fig. S4I, J). Hence, we identified E_C4 as terminally differentiated keratinocytes in ACP. The process of keratinization in keratinocytes is required to produce abundant proteins and lipids to construct the cornified envelope, strong activation of lipid metabolic pathways in E_C4 also proved our hypothesis (Fig. S3D) [51]. The E_C5 cluster, which had a similar transcriptome to E_C1, was identified by cell cycle genes such as MKI67 and STMN1, illustrating a proliferating state (Fig. 4C).

To further evidence our analysis results, we first evaluated the expression program in these subclusters according to the literature (Fig. 4I) [52]. We observed that the cycling program was primarily activated in E_C1/E_C2 clusters, which meant that these two subclusters had a high proliferative potential or ability to regulate the proliferation of other cells. RNA velocity showed strong directional streams from E_C2 to other subclusters, predicting it as the origin of ACP epithelial cells (Fig. 4J). We also found a directional stream from E_C5 to E_C1, hinting that the E_C5 cluster was probably a proliferative fraction of E_C1 (Fig. 4J). Expression program patterns were more similar between the E_C3 and E_C4 clusters. However, compared to E_C3, E_C4 had a stronger activated Epi2 program (represented terminal differentiation); a higher score for Epi1 program (represented expression of keratins); and an upregulation of programs composed of mucosal program and stress program (all related to the apoptosis pathway) (Fig. 4I). Similarly, RNA velocity also revealed that the E_C4 cluster was one end point of ACP epithelial cell differentiation (Fig. 4J).

We noticed that the E_C3 cluster (KCs) had a high level of activation of epithelial-mesenchymal transition (EMT). Thus far, no such process inside the tumour has been reported. It led us to wonder whether keratinocytes in ACP undergo the process of EMT. IHC staining was performed with S100A4, a marker of fibroblasts produced by EMT [53]. We found that the tumour parenchyma, especially near keratins, showed positivity of S100A4. Its colocalization with pan-keratin was confirmed by mIHC staining (Fig. 4K), indicating the existence of partial EMT (pEMT). The keratinization of keratinocytes, which was also considered the differentiation trajectory of the E_C4 cluster, is a unique process of cell death that is controlled by a complex network of TFs. To determine the major TFs of E_C4, we performed pySCENIC. We found that EHF and ELF3, both of which are ETS family members, were highly expressed and activated, which may represent the major TFs that drive this differentiation trajectory (Fig. 4L–N). In short, our results identified and characterized 5 ACP cell types by deciphering intratumoral heterogeneity. Activation of pEMT was also found in ACP keratinocytes, suggesting a potential migratory mechanism. Moreover, we identified one type of terminally differentiated keratinocyte that could participate in tumour immunity.

### Spatial transcriptomics reveals spatial features of various subclusters in the ACP and colocalization of the TAM_GPNMB and E_C4 clusters

**Fig. 5:**
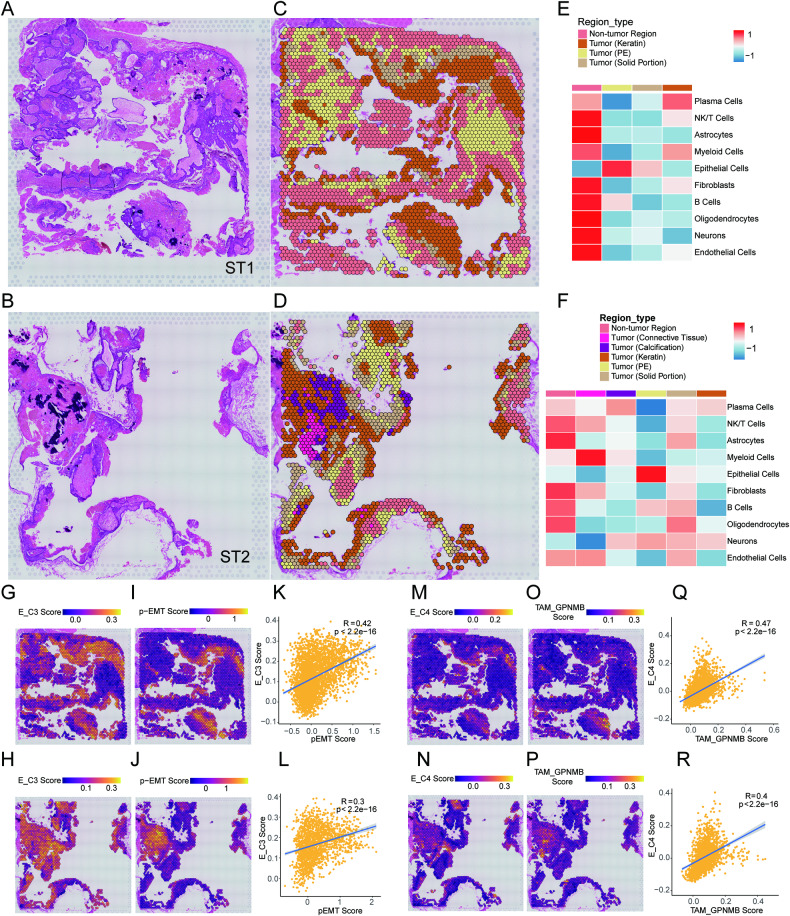
Spatial visualization of different cell types from snRNA-seq data. **A**, **B** H&E staining images of sections showing the distinct pathological features of ST1 and ST2 samples. **C**, **D** Spatial spots are separated into different regions with distinct pathological features through unbiased clustering in ST1 and ST2 samples. **E**, **F** Heatmaps showing the mean signature scores of 10 cell types in each divided region. **G**, **H** Signature scores of the E_C3 cluster in each spot on ST sections from ST1 and ST2 samples. **I**, **J** Scores of the pEMT signature in each spatial spot on ST sections from ST1 and ST2 samples. **K**, **L** The correlations for signature score of pEMT and E_C3 cluster in each spatial spot from ST1 and ST2 samples, respectively. The error band shows the 95% confidence interval. **M, N** Signature scores of the E_C4 cluster in each spot on ST sections from ST1 and ST2 samples. **O**–**P** Signature scores of the TAM_GPNMB cluster in each spot on ST sections from ST1 and ST2 samples. **Q**, **R** The correlations for the signature scores of the TAM_GPNMB and E_C4 clusters in each spatial spot from the ST1 and ST2 samples.

To further characterize the spatial distribution of different cell types in ACP, ST was performed on ACP tissue sections. We mounted 4 tissue samples from 3 patients on the spatially barcoded ST microarray slides (Table S1). Using unbiased clustering, spatial spots from different regions could be separated from each other in all sections (Fig. S5A). All ST samples had characteristic pathologic features of typical ACP (Fig. 5A, B and S5B, C).

Then, we assessed the overall cell distribution in different regions of the sections by calculating the nucleus subcluster signature score in each spot based on our snRNA-seq data (top 100 specifically expressed genes) (Fig. 5C–F and S5D–G). The low infiltration of NK/T cells can be clearly visualized within the ACP parenchyma, and there was a significant negative correlation between the signature score of NK/T cells and epithelial cells, showing a repulsion relationship between these two subclusters (Fig. S5H). Based on the same method, we also examined the spatial features of epithelial cell subclusters. The distribution of epithelial subclusters revealed by respective signature score was basically consistent with our forecast (Fig. S6A, B). Among them, the signature score of E_C3 cluster, which was located in solid portion and near the clumps of keratin, and pEMT was significantly positively correlated (Fig. 5G–L and S6C–E) [54]. We also observed that the pEMT score was higher closer to the keratins, further corroborating the tight link between KC migration and pEMT. The terminally differentiated keratinocyte E_C4 cluster was visualized within the clumps of keratin, which also conformed our identification (Fig. 5M, N and S6F). The highly proliferative E_C5 cluster was located mostly at PE close to the edge between hypothalamus and ACP (Fig. S6G). Therefore, ST data support our analysis of snRNA-seq data.

Interestingly, some spatial spots with high E_C2 signature score were near highlighted spots with E_C4 signature, hinting that some of WCs might have close spatial relationship to terminally differentiated keratinocytes. This was corroborated by mIHC staining, which showed that WCs with nucleo-cytoplasmic β-catenin accumulation and nearby RHCG^+^ terminally differentiated keratinocytes were both positive for CDKN2A, suggesting the senescence association with this differentiation process (Fig. S7A, B). Moreover, we compared the signature scores of senescence associated secretory phenotype (SASP) and E_C2/E_C4 in ST sections (Fig. S7C, S7E, S7G and S7I). The results revealed that E_C2 and E_C4 signature spatially both correlated with SASP signature, which further emphasized the important role of cellular senescence in the development of ACP (Fig. S7D, S7F, S7H and S7J).

**Fig. 6:**
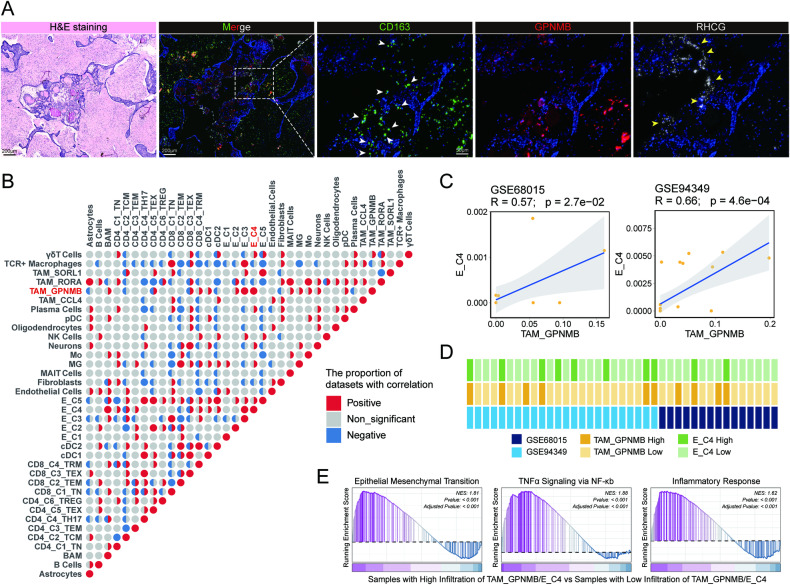
Spatial colocalization and infiltration correlation of TAM_GPNMB and the E_C4 cluster. **A** asmFISH of ACP tissues with keratins, showing the close spatial relationship between TAM_GPNMB and the E_C4 cluster. CD163 (green), GPNMB (red) and RHCG (white) are shown in individual channels respectively. H&E-stained adjacent section to show histology. The white arrows indicate TAM_GPNMB cells. The yellow arrows indicate E_C4 cells. Scale bar: 200 μm (left one, two), 200 μm (right one, two and three). **B** Pie charts showing the proportion of datasets with different correlation for infiltration of paired cell subtypes (Positive in red was defined as correlation coefficient [*R*] > 0.3; negative in blue was defined as *R* < −0.3; non-significant in grey). **C** The correlations for the infiltration of TAM_GPNMB and the E_C4 cluster in each dataset with ACP, including GSE68015 (*n* = 15; *R* = 0.57; *p* = 2.7e−02) and GSE94349 (*n* = 24; *R* = 0.66; *p* = 4.6e−04). The error band shows the 95% confidence interval. **D** The characteristics of cell infiltration in datasets. High infiltration is defined as the top twenty-five percent of infiltration group. **E** Enriched signature of epithelial mesenchymal transition, TNFα signalling via NF-κb and inflammatory response in group with high infiltration of TAM_GPNMB and E_C4 by GSEA.

Considering the functional crosstalk between the TAM_GPNMB and the E_C4 cluster, we compared the signature scores of TAM_GPNMB/E_C4. The results underscored the colocalization of TAM_GPNMB and E_C4 in the same spatial spot (Fig. 5O, P and S8A). Moreover, there was a most significant positive correlation between the signature score of these two clusters (Fig. 5Q, R and S8B–F). By asmFISH, we confirmed the close spatial relationship between these two types of subclusters near keratins in ACP (Fig. 6A). As the spatial distribution demonstrated a high correlation between TAM_GPNMB and the E_C4 cluster, we calculated the Spearman correlations for the proportion of cell infiltrations evaluated by CIBERSORTX in a larger cohort from the Gene Expression Omnibus (2 datasets with 39 ACP samples). We noticed that there was the most highly positive correlation between TAM_GPNMB and the E_C4 cluster in two datasets, which were all significantly (Fig. 6B, C). The colocalization and correlation of infiltration illustrated an underlying interaction between these two cell types. To further uncover the potential influence arising from TAM_GPNMB and the E_C4 cluster, 39 ACP samples in datasets were divided into two groups based on the TAM_GPNMB/E_C4 infiltration level (one group was with high infiltration of TAM_GPNMB and E_C4, the other group was with the rest) (Fig. 6D), and we compared the DEGs between two groups subsequently. GSEA showed an upregulation of EMT signature in samples with high level of infiltration (Fig. 6E), which is consistent with the assumed effect that the crosstalk between these two type cells was associated with tumour progression. These samples also exhibited enrichment of TNFα/NF-κb signalling and inflammatory response signatures, suggesting the existence of environmental stimulus or highly activated immune response (Fig. 6E).

### Intercellular interaction between TAM_GPNMB and ACP epithelial cells

To determine the key targets of the E_C4 cluster and TAM_GPNMB interaction in ACP and to investigate the involvement of TAM_GPNMB in the regulation of E_C4 differentiation, considering the positive correlation of infiltration between TAM_GPNMB and E_C3/E_C4 clusters and the spatial proximity of these two epithelial subclusters, we explored the intercellular interaction pathways within the TAM_GPNMB and E_C3/E_C4 clusters.

**Fig. 7:**
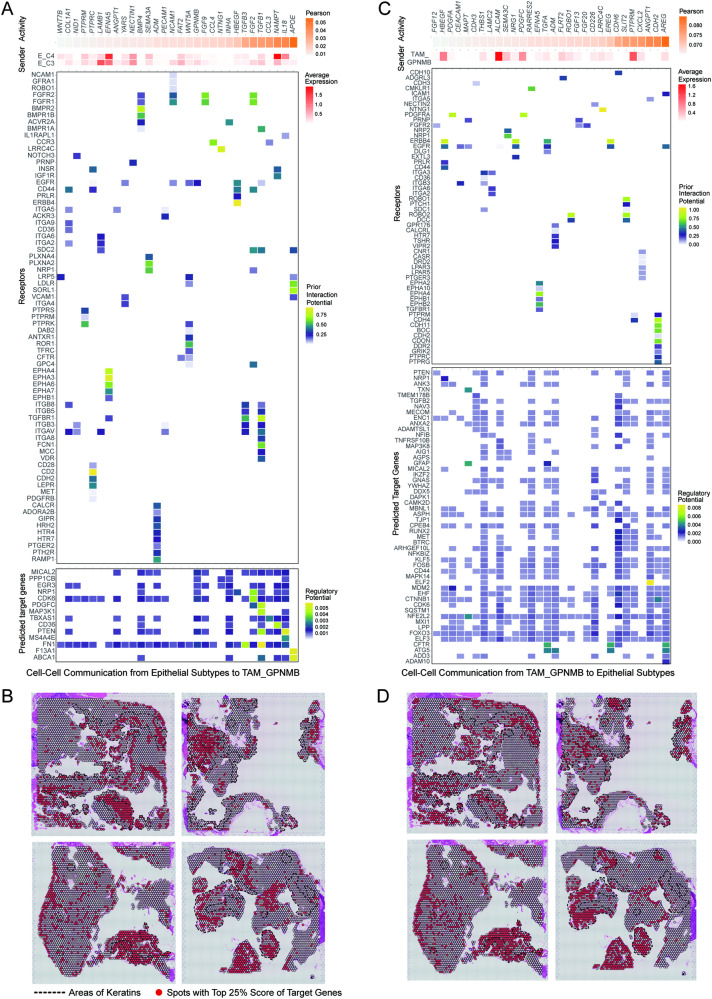
Intercellular communication network between TAM_GPNMB and epithelial cell subclusters as determined by Nichenet. **A** Heatmaps showing ligand‒receptor communication streams from E_C3/E_C4 clusters to TAM_GPNMB. Top, orange heatmap indicates the Pearson correlation of prioritized ligands from high to low, and red heatmap indicates the expression level of ligands in E_C3/E_C4 clusters. Middle, heatmap of ligand‒receptor pairs, coloured by interaction potential. Bottom, heatmap showing the ligand and predicted target genes, coloured by regulatory potential. **B** Spatial sections, named ST1 to ST4 samples, showing spatial spots with the top 25% signature score of predicted target genes in TAM_GPNMB which were colour-coded in red. Signature scores were calculated by the AddModuleScore function in Seurat. The black dotted lined areas represent keratins. **C** Heatmaps showing ligand‒receptor communication streams from TAM_GPNMB to E_C3/E_C4 clusters. **(D)** Spatial sections showing spatial spots with the top 25% signature score of predicted target genes in E_C3/E_C4 clusters which were colour-coded in red. The black dotted lined areas represent keratins.

Ligand genes in E_C3/E_C4 clusters had similar expression levels (Fig. 7A). We found that E_C3/E_C4 clusters could recruit TAM_GPNMB through the ligand‒receptor pairs CCL3/CCL4-CCR3 (Fig. 7A) [55]. Moreover, with the expression of TGF-β family genes, including TGFB1, TGFB3 and HBEGF, these two subclusters could induce the involvement of TAM_GPNMB in extracellular matrix remodelling, thereby promoting the migration of KCs (Fig. 7A) [56–58]. Significantly, we prioritized the top ligands and observed that the ligand‒receptor pairs NAMPT-IGF1R and APOE-LDLR were highly active (Fig. 7A). Reportedly, inducing NAMPT can increase steroidogenesis through the induction of IGF-1, and the APOE-LDLR pair can reduce macrophage apoptosis by improving lipid metabolism, suggesting that the existence of E_C3/E_C4 clusters mediates the lipid phenotype conversion of TAM_GPNMB [59, 60]. By scoring the predicted target gene set on ST, we observed the enrichment of the target gene set mostly in the region with keratins and nearby (Fig. 7B), and the function of which may focus on the regulation of cell-substrate adhesion, the metabolism of lipids and interleukin-4 and interleukin-13 signalling (Fig. S9A). It was consistent with the putative results according to ligand‒receptor communications, indicating that E_C3/E_C4 clusters could affect TAM_GPNMB in several ways, and finally causing tumour progression [61].

Ligand genes, such as AREG, EREG, EGFR and TGFA, were found to be highly active in TAM_GPNMB, suggesting that TAM_GPNMB may promote KC migration through these ligand pathways (Fig. 7C) [62–65]. Meanwhile, ligand‒receptor pairs related to EMT, including CXCL12-CASR and PDGFC-PDGFRA, were observed between TAM_GPNMB and E_C3/E_C4 clusters (Fig. 7C) [66, 67]. Interestingly, TAM_GPNMB showed the potential to block the translocation of β-catenin into the nucleus, weaken cell stemness, promote terminal differentiation through the ligand‒receptor pairs SLIT2-ROBO1 and FGF20-FGFR2. TAM_GPNMB could also induce programmed cell death via the expression of the ligand EFNA5, which supported keratinization (Fig. 7C) [68–71]. The enrichment analysis for predicted downstream target genes coincided with the analysis of ligand‒receptor pairs mentioned above (Fig. S9B). The target genes were also found to be expressed in the region full of keratins and nearby (Fig. 7D). In conclusion, our study reveals an interaction network built by TAM_GPNMB and E_C3/E_C4 clusters, which can maintain each other’s function and presence. All these findings emphasize the significance of TAM_GPNMB in shaping the specific TME of ACP.

## Discussion

Our results demonstrate that ACP has a typically immunosuppressive microenvironment, with low infiltration of lymphocytes in the solid portion. As one kind of central nervous system neoplasm, we identified MG and BAM in ACP as potential sources of TAM along with Mo. Among all subtypes of TAM, we focused on TAM_GPNMB, which is a class of non-microglial-derived TAM related to lipid metabolism and with a greater propensity towards M2 polarization. GPNMB, which is localized on the cell surface or stored in lysosomes, is one kind of highly glycosylated transmembrane protein [72]. It is highly expressed in many other tumours, like glioma and melanoma [73, 74]. The extracellular domain of GPNMB is found binding to several receptors on tumour cells. And its intracellular domain benefits to macrophage activity through autocrine effects [75]. With the expression of GPNMB in macrophages, there were a few studies that described the increased production of anti-inflammatory cytokines and the inhibition of T-cell responses [76]. In glioblastoma, GPNMB-high macrophages can induce mesenchymal transformation [77]. Moreover, the soluble GPNMB from macrophages can promote cancer cell survival and expansion [72]. Our study further demonstrated the significance of TAM_GPNMB in tumour development. The localization of TAM_GPNMB in regions that enriched keratins raises an intriguing question about the role of keratin in influencing macrophage polarization. M2 phenotype conversion induced by keratin has been reported, but the underlying mechanism is still unknown [78, 79]. Although pySCENIC showed high ATF3 activity that skews macrophages towards an M2 phenotype, we still need more evidence to determine the key TFs of TAM _ GPNMB.

In this study, we systemically deciphered the heterogeneity of epithelial cells and identified 5 subclusters that could represent characteristic pathologic features in ACP. E_C2, representing WCs with nucleo-cytoplasmic accumulation of β-catenin, was identified in our data. Consistent with the work from J. Apps et al, E_C2 secreted multiple cytokines [11]. We further found that there is heterogeneity in this cluster, and different E_C2 subclusters had different secretion patterns. Although Jiang et al suggested that ACP cells might start from PE [80], WC was characterized with high differentiation potential according to our snRNA-seq data, and was predicted to be the origin of differentiation trajectories, which is consistent with the role played by senescence in the activation of pathways related to the cancer stem cell phenotypes, and the role played by the WNT signalling pathway in the maintenance of cell stemness [81, 82]. And by cell sorting and culturing, Wang et al found that ACP cells with nuclear translocation of β-catenin had multidifferentiation potential, which as well substantially matched our results [37]. The E_C3 and E_C4 clusters, which are closely related to the formation of keratin, have distinct expression patterns of keratin genes. Reorganization of the keratin cytoskeleton owing to this difference promotes epithelial cell migration [4]. This redistribution of the keratin network may result in the enlargement of specific keratin clumps in ACPs and then lead to tumour growth. We found TAM_GPNMB was spatially colocalized with the E_C4 cluster in tumours, the interaction between these two might promote keratinization in various ways. The E_C4 cluster is a group of terminally differentiated keratinocyte in the keratinization status. We identified it by a high level of CDKN2A, but Gonzalez-Meljem et al had found the positive CDKN2A staining in WC [50]. This could be attributable to the existence of differentiation relationship from E_C2 to E_C4. But it also means the widespread senescence within the ACP TME, consistent with Prince et al. recent work, further emphasizes the importance of senescence in ACP development [83]. By performing RNA velocity analysis, this cluster was predicted as one terminus of the differentiation trajectory. TFs that belong to the ETS family, namely, EHF and ELF3, may play key regulatory roles in this differentiation process [84, 85]. EHF/ELF3 could suppress cell stemness and was associated with keratinocyte differentiation. EHF has been reported as a therapeutic target of rosiglitazone in pancreatic cancer [86], indicating a promising target for ACP in the future. For now, targeted therapies for ACP remain under exploration. While WNT pathway highly activates in tumour, off-target effect led to unsatisfactory result of the use of related inhibitors. The activation of the MAPK/ERK pathway in the leading edge of tumour unveiled its therapeutic potential, and related clinical trials are currently underway [87]. The presence of cellular senescence in many regions makes it possible to target senescence in ACP. However, related clinical evidence is still lacking. We here provide new insight into the targetable pathway of ACP, with the expectation to use it clinically. In short, our analyses characterized the heterogeneity of epithelial cells in ACP. We highlighted the function of distinct epithelial subtypes and clearly confirmed the critical role that the E_C4 cluster plays in the process of keratinization preliminarily. Our work formulated a hypothesis that the interaction between TAM_GPNMB and E_C4 should be considered a therapeutic target for ACP, as this could delay tumour progression.

We noted that the predicted downstream target genes that acted on E_C4 were enriched for terms related to atherosclerosis. Close to high score spatial spots with TAM_GPNMB and/or E_C4 cluster signatures, calcification was frequently observed. This finding reminds us of the functional similarity of TAM in ACP to that of macrophages reported in atherosclerosis. Atherosclerosis is a lipid-enriched microenvironment in which macrophages undergo foam cell transformation [88]. This subpopulation of macrophages has the capacity for efferocytosis, which can clear the necrotic core in atherosclerotic plaques, similar to BAM identified by our data in ACP. However, excessive LDL uptake induces macrophage apoptosis, similar to TAM_RORA in ACP. The existence of necrotic core, a hallmark of vulnerable plaques in atherosclerosis, is associated with impaired clearance by macrophages in diseased blood vessels [89, 90]. Interestingly, TAM_GPNMB, which was less capable of efferocytosis, showed spatial colocalization with the terminally differentiated cell subcluster (E_C4). We reasonably surmised that the ghost cell formed after E_C4 cluster apoptosis might be similar to the necrotic core in atherosclerosis. And TAM_GPNMB with impaired efferocytosis might not clear E_C4 cluster, instead it will eventually form large calcification clumps following interactions with microcalcification clusters secondary to apoptotic components. The large calcification clumps could affect the conversion of macrophages to the M2 phenotype and inhibit osteoclast differentiation to further promote this process [91].

In conclusion, our study provides a comprehensive picture of the landscape of the immune microenvironment, and unveils the detailed landscape of both immune and epithelial cells in ACP. Our findings imply that ACP is one kind of immune-excluded tumour, and the interactions between one terminally differentiated subpopulation of epithelial cells that highly express RHCG and TAM_GPNMB play a key role in regulating the microenvironment to promote keratinocytes migration and differentiation. We provide a novel insight for targeting immunotherapy by intervening in specific cell subtypes in ACP. The lack of mature cell lines and the difficulty in ACP cell culture after sorting limits the progress of our study, and all interaction mechanisms still need to be confirmed in future work.

## Materials and methods

### ACP sample collection

ACP specimens were collected from the Department of Neurosurgery of the First Affiliated Hospital of Nanchang University. All patients were diagnosed with ACP after postoperative pathological examination (patient information is shown in Table S1). For snRNA-seq and ST assays, surgical specimens were divided into the following two classes: tumour tissues with adjacent hypothalamus and tissues away from the hypothalamus (based on intraoperative labelling); the samples were then quick-frozen in liquid nitrogen and finally stored at −80 °C. This study was approved by the Institutional Review Committee of the First Affiliated Hospital of Nanchang University with the informed consent of patients.

### Nucleus isolation

Thawed ACP specimens were chopped into small pieces on ice and transferred into a 1.5 mL tube with 1 mL of chilled Nuclei EZ Lysis Buffer. Specimens homogenized by a douncer with 20 strokes were transferred into a 2 mL tube with 1 mL of chilled Nuclei EZ Lysis Buffer. After a 5 min incubation on ice, the homogenate was filtered through a 70 μm-strainer mesh and centrifuged at 500 × *g* for 5 min at 4 °C. After the supernatant was carefully removed, nuclei were resuspended gently in another 1.5 mL of EZ lysis buffer. After another 5 min incubation on ice and subsequent centrifugation (500 × g for 5 min at 4 °C), the supernatant was removed, and then 500 µL Nuclei Wash and Resuspension Buffer were added immediately. Finally, after repeated incubation, centrifugation and addition steps, all nuclei were collected for further experiments.

### Library preparation

The prepared single nuclei suspension was loaded on the Chromium Controller (10× Genomics) to prepare libraries using the Chromium Single Cell 3ʹ Reagent Kits v3 (10x Genomics) according to the manufacturer’s instructions. In brief, after a series of experimental steps, including cell counting and quality control, gel bead-in-emulsion (GEM) generation and barcoding, post-GEM-RT cleanup and cDNA amplification, libraries were prepared and sequenced on a NovaSeq platform (Illumina).

### Quality control of snRNA-seq data

Raw sequencing files were processed and mapped to the reference genome (GRCh38) by the 10X Genomics CellRanger pipeline using default parameters. Unique molecule identifiers (UMIs) were counted to create gene expression matrices, and we used the R package Seurat v3 for data filtering to obtain a high-quality nucleus [92]. The nuclei filtering thresholds were determined by UMI counts, expressed gene number, percentage of mitochondrial gene counts and ribosomal gene counts.

### Dimension reduction and clustering analysis of snRNA-seq data

After filtering out low-quality nuclei, gene expression in each nucleus was normalized, and data from different samples were integrated using the default workflow of SCTransform [93]. To mitigate the effect of unwanted sources of variation, we also scaled the data by the regression of cell cycle influence and the percentage of mitochondrial gene counts in each nucleus. PCA was performed with the top 3000 most variable genes, and nucleus clusters were obtained using the FindNeighbors and FindClusters functions and visualized with UMAP. Finally, clusters were identified according to the reported cell type marker genes. In addition, nuclei enriched in the expression of marker genes for multiple lineages were excluded from further analyses.

### Differentially expressed genes and functional enrichment analysis

The built-in FindAllMarkers function in Seurat was used to detect DEGs for each nucleus cluster with the following parameters: min.pct = 0.25, logfc.threshold = 0.25, and only.pos = TRUE. For snRNA-seq data, hallmark gene sets from MSigDB were used to perform gene set variation analysis, which was implemented by the GSVA package [94, 95]. We also used differentially expressed genes identified in each nucleus cluster, which were detected as previously mentioned, to perform functional enrichment analysis and PPI network analysis through Metascape, an integrated and user-friendly web tool [96]. Cytoscape was used to visualize the PPI network. To evaluate the expression of the gene signature set of IRF8 in TAMs, we first extracted ACP samples and normal brain samples from the datasets GSE94349 and GSE68015, which were obtained from the Gene Expression Omnibus database, and divided them into two groups [12, 13]. Then, we used GSEA to assess whether there was significant enrichment of the IRF8 gene signature set in differentially expressed genes of ACP compared to normal brain [97, 98].

### Digital cytometry using CIBERSORTx

Digital cytometry was carried out to establish the proportions of cell types defined by our snRNA-seq data in larger cohorts from microarray data. Using the online CIBERSORTx, we generated a reference matrix consisting of subdivided cell types from our snRNA-seq data [99]. To avoid memory errors, we limited the size of the reference matrix by randomly down-sampling to a maximum of 15000 nuclei. Then, we used this reference matrix to create a signature matrix through the default settings. Microarray mixture datasets were analysed with quantile normalization performed prior to deconvolution.

### Similarity analysis

To explore the similarity between different cell types, we used Pearson correlation calculated based on the mean expression levels of 1500 highly variable genes of nuclei in each cluster. The results were visualized in heatmaps.

### Definition of functional scores

To illustrate the functional properties of different cell types, we collected sets of genes from the literature and calculated functional scores. For NK/T cells, cytotoxic genes were collected from Zheng et al. [100]; exhausted genes were collected from Guo et al. [20]; and effector genes were from Herbst et al. [21]. For myeloid cells, the genes for foam cells were defined as in Kim et al. [38]; efferocytosis-associated genes were extracted from related literature (Kojima et al. [90]; Myers et al. [101]; Boada-Romero et al. [89].); inflammasome genes were collected from the integration of related literature [102–105]; apoptosis-related genes were adapted from “Apoptosis (KEGG PATHWAY: hsa04210)”; signature genes for macrophage polarization were defined as in He et al. [40]; and genes to distinguish monocyte-like TAM from microglia-like TAM were collected from Ochocka et al.. IRF8 signature genes were from Nixon et al. [29]. Eight expression program scores of epithelial cells were calculated with gene sets from Zhang et al. [52]. Genes for SASP signature were adapted from “Senescence-Associated Secretory Phenotype (REACTOME: R-HSA-2559582)”.

For the scores shown in the heatmaps, the mean expression level of each cell cluster was used to calculate the score by the GSVA package. For the scores shown in the violin diagrams, we used the AddModuleScore function in Seurat to calculate signature scores. The genes for each score are listed in Table S4.

### Trajectory inference based on RNA velocity estimation

To investigate the correlation of differentiation of subdivided cell types, the bam files generated by Cell Ranger were imported into the Velocyto pipeline to create loom files recounting the spliced and unspliced reads. scVelo in Python was used to perform RNA velocity analysis based on loom files [106]. The velocities were projected onto UMAP and visualized as streamlines using the scv.pl.velocity_embedding_stream() function.

### Analysis of transcription factor regulon

The regulon activity was evaluated by pySCENIC in Python for different main cell types [107]. In brief, the regulon activity was analysed by the AUCell module in pySCENIC for each TF regulon. The regulon activities and TF expression were visualized using the FeaturePlot function in Seurat or shown in heatmaps.

### Cell‒cell communication

The NicheNet package was used to infer ligand‒receptor interactions among subpopulations separately based on snRNA-seq data [108]. For ligand‒receptor interactions, genes expressed in more than 10% of cell clusters were considered. Ligand activity was ranked using the Pearson test. The top 30 ligands of sender cells were extracted for subsequent ligand‒receptor and ligand-target network analysis. All interaction networks were visualized based on the official pipeline.

### Experimental procedure of spatial transcriptomics

All four ACP tissues (two with adjacent hypothalamus and two away from the hypothalamus) were snap-frozen with precooled isopentane and then embedded with Optimal Cutting Temperature (OCT) compound to preserve the structure of tissues. Cryosections were cut from the OCT-embedded tissues and placed on Visium Spatial Slides for tissue sections. After RNA quality assessment, tissue optimization and permeabilization, RT Master Mix containing reverse transcription reagents was added to the permeabilized tissue sections for cDNA synthesis. At the end of first-strand synthesis, second-strand Mix was added to the tissue sections on the slide, followed by cDNA amplification and library construction. Sequencing was conducted by the Illumina NovaSeq6000 platform.

### Processing and analysis of spatial transcriptomic data

Sequencing read alignment, filtering, barcode counting, and UMI counting of spatial transcriptomic data were performed separately for each section using Space Ranger v1.1. The gene-spot matrices were analysed in R using Seurat V3, and SCTranform was used for data normalization. Dimension reduction and clustering were performed with PCA at a suitable resolution for each section. Signature scoring derived from snRNA-seq signatures, the target gene set of cell interactions, and pEMT signature genes from Pal et al. (refer to Table S4) was performed with the AddModuleScore function for each spot [54]. Spatial expression feature plots were generated with the SpatialFeaturePlot function in Seurat.

### Multiplex immunohistochemical (mIHC) and immunohistochemical (IHC) staining

ACP tissues from surgery were fixed in 4% paraformaldehyde over 24 h. After dehydration, we embedded the samples in paraffin to section them. The sections were deparaffinized and rehydrated with graded alcohol concentrations. We next immersed the sections in EDTA antigen retrieval buffer (pH 8.0) while placing them in a microwave oven to retrieve antigen. For mIHC, endogenous peroxidase and 3% BSA were used for blocking. Sections for mIHC staining were incubated with the primary antibody and secondary antibody sequentially according to the manufacturer’s protocols. After incubation with the corresponding solution for tyramide signal amplification, sections were immersed in EDTA antigen retrieval buffer (pH 8.0) again to remove the primary antibodies and secondary antibodies combined with tissues. Subsequently, sections were subjected to the next round of staining and treatment until all markers were stained. For IHC, after antigen repair, sections were blocked by incubating with 3% hydrogen peroxide and washed with PBS. Then sections were blocked with 5% BSA for 30 min at 25 °C and incubated with primary antibodies at 4 °C overnight. After washing with PBS, sections were incubated with secondary antibody at room temperature for 50 min. After DAPI counterstaining in the nucleus, we scanned sections and visualized images in CaseViewer. All antibody information is listed in Table S5.

### Fluorescence in situ hybridization (FISH) and amplification-based single-molecule fluorescence in situ hybridization (asmFISH)

FISH for RSPO2 was performed on paraffin-embedded sections by Servicebio Company (Wuhan, China). After hybridization with probe, sections were counterstained in DAPI. asmFISH was performed on paraffin-embedded tissue sections by Suzhou Dynamic Biosystems Co., Ltd (Suzhou, China). The operation process can be summarized as follows. After baking and deparaffinization, sections were dehydrated with a gradient of ethanol and then were permeabilized. When sections were processed, using probe hybridization and rolling circle amplification, asmFISH was conducted. Finally, image collection was performed with a Leica DM6B microscope (Leica, Germany).

### Statistical analysis

All data were performed normality testing. The Wilcoxon rank-sum test was conducted using R as appropriate. *P*-values < 0.05 were considered statistically significant.

### Supplementary information

Figures S1-9

Table S1

Table S2

Table S3

Table S4

Table S5

## Data Availability

The data that support the findings of this study are available in GSE215932. Any additional data required are available from the corresponding author upon reasonable request.
