# Supplementary material for: Multiomics integration-based immunological characterizations of adamantinomatous craniopharyngioma in relation to keratinization
Source: Cell Death Dis. 2024 Jun 21;15(6):439. doi: 10.1038/s41419-024-06840-1 (PMC11192745; doi:10.1038/s41419-024-06840-1)
Supplement: Supplementary file 1 — Figures S1-9 [file 41419_2024_6840_MOESM1_ESM.pdf]

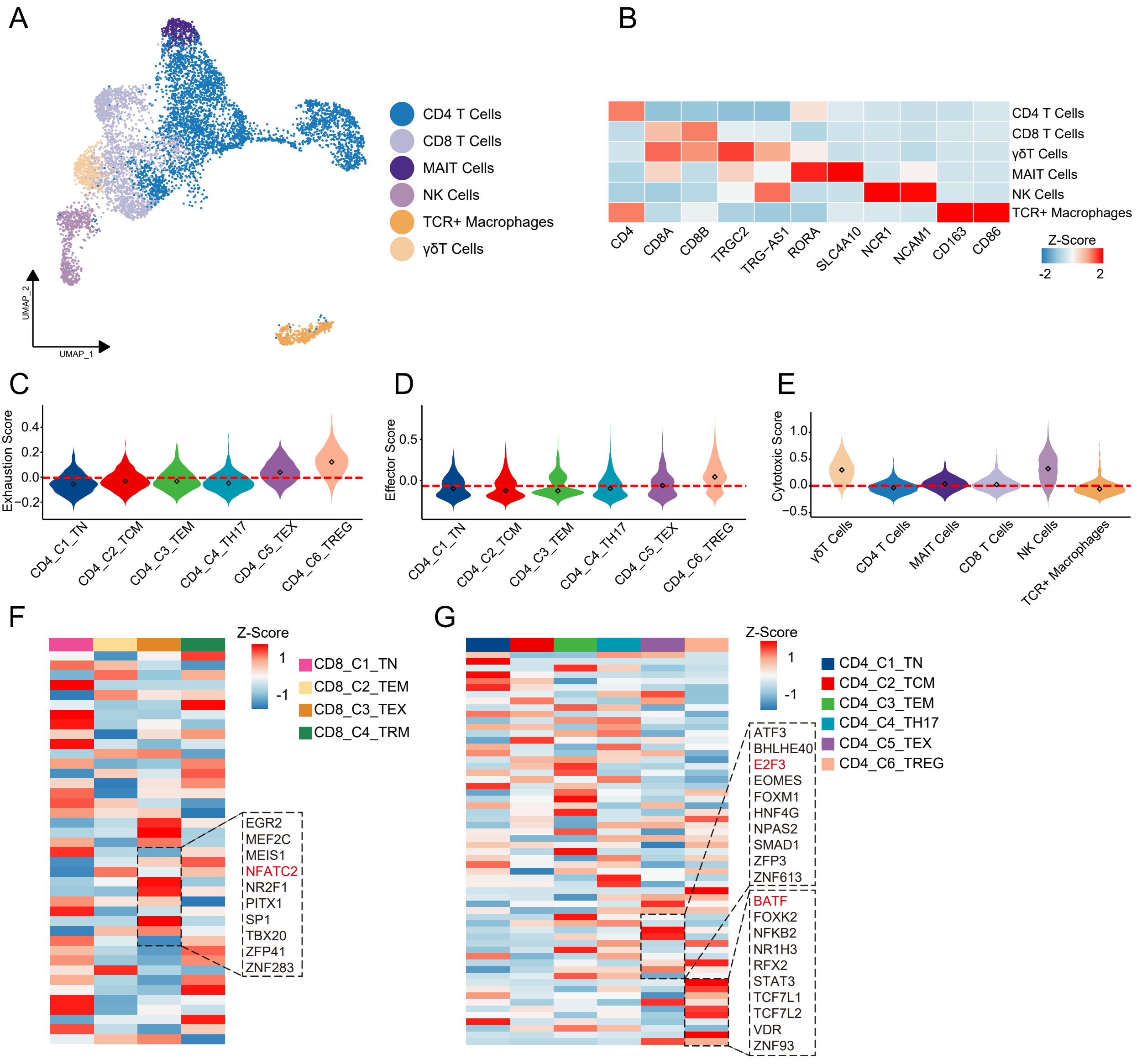

A
CD4 T Cells
CD8 T Cells
MAIT Cells
NK Cells
TCR+ Macrophages
γδT Cells
UMAP_1
UMAP_2
B
CD4 T Cells
CD8 T Cells
γδT Cells
MAIT Cells
NK Cells
TCR+ Macrophages
CD4
CD8A
CD8B
TRGC2
TRG-AS1
RORA
SLC4A10
NCR1
NCAM1
CD163
CD86
Z-Score
-2
2
C
Exhaustion Score
0.4
0.2
0.0
−0.2
CD4_C1_TN
CD4_C2_TCM
CD4_C3_TEM
CD4_C4_TH17
CD4_C5_TEX
CD4_C6_TREG
D
Effector Score
0.5
0.0
CD4_C1_TN
CD4_C2_TCM
CD4_C3_TEM
CD4_C4_TH17
CD4_C5_TEX
CD4_C6_TREG
E
Cytotoxic Score
1.0
0.5
0.0
−0.5
γδT Cells
CD4 T Cells
MAIT Cells
CD8 T Cells
NK Cells
TCR+ Macrophages
F
Z-Score
1
-1
CD8_C1_TN
CD8_C2_TEM
CD8_C3_TEX
CD8_C4_TRM
EGR2
MEF2C
MEIS1
NFATC2
NR2F1
PITX1
SP1
TBX20
ZFP41
ZNF283
G
Z-Score
1
-1
CD4_C1_TN
CD4_C2_TCM
CD4_C3_TEM
CD4_C4_TH17
CD4_C5_TEX
CD4_C6_TREG
ATF3
BHLHE40
E2F3
EOMES
FOXM1
HNF4G
NPAS2
SMAD1
ZFP3
ZNF613
BATF
FOXK2
NFKB2
NR1H3
RFX2
STAT3
TCF7L1
TCF7L2
VDR
ZNF93

Figure S1. Immunological features of NK/T cells in ACP. Related to Figure 2.

(A) UMAP of NK/T cells, showing 6 subclusters in different colours. All subclusters are labelled on the right. **(B)** Heatmap of marker genes for NK/T cells, coloured by the expression of the genes. The expression levels are z score normalized in the heatmap. **(C-E)** Violin plots showing the exhaustion and effector score in $CD4^+$ T cells, and the cytotoxic score in NK/T cells. The dashed line indicates the median signature score, and the rhombus point in each violin represents its own median score. **(F-G)** Heatmaps showing the expression of TFs, which are consistent with Figure 2J-K for each subcluster in $CD8^+$ T cells and $CD4^+$ T cells, respectively. The expression is z score normalized. The dotted black boxes indicate the top 10 regulons in specified subcluster, only the regulons of interest are labelled in red.

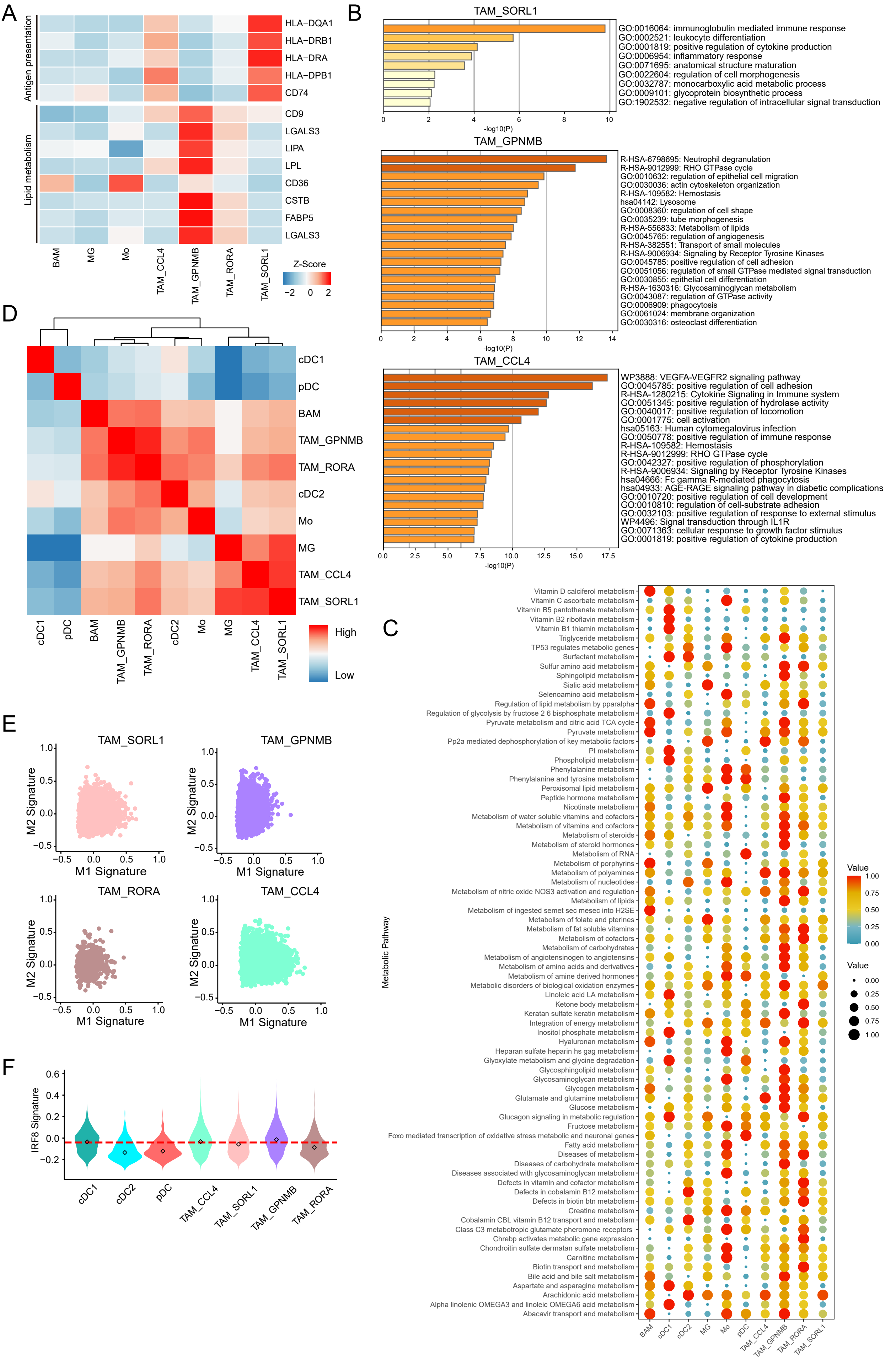
A
Antigen presentation
Lipid metabolism
HLA−DQA1
HLA−DRB1
HLA−DRA
HLA−DPB1
CD74
CD9
LGALS3
LIPA
LPL
CD36
CSTB
FABP5
LGALS3
BAM
MG
Mo
TAM_CCL4
TAM_GPNMB
TAM_RORA
TAM_SORL1
Z-Score
−2 0 2
B
TAM_SORL1
GO:0016064: immunoglobulin mediated immune response
GO:0002521: leukocyte differentiation
GO:0001819: positive regulation of cytokine production
GO:0006954: inflammatory response
GO:0071695: anatomical structure maturation
GO:0022604: regulation of cell morphogenesis
GO:0032787: monocarboxylic acid metabolic process
GO:0009101: glycoprotein biosynthetic process
GO:1902532: negative regulation of intracellular signal transduction
-log10(P)
TAM_GPNMB
R-HSA-6798695: Neutrophil degranulation
R-HSA-9012999: RHO GTPase cycle
GO:0010632: regulation of epithelial cell migration
GO:0030036: actin cytoskeleton organization
R-HSA-109582: Hemostasis
hsa04142: Lysosome
GO:0008360: regulation of cell shape
GO:0035239: tube morphogenesis
R-HSA-556833: Metabolism of lipids
GO:0045765: regulation of angiogenesis
R-HSA-382551: Transport of small molecules
R-HSA-9006934: Signaling by Receptor Tyrosine Kinases
GO:0045785: positive regulation of cell adhesion
GO:0051056: regulation of small GTPase mediated signal transduction
GO:0030855: epithelial cell differentiation
R-HSA-1630316: Glycosaminoglycan metabolism
GO:0043087: regulation of GTPase activity
GO:0006909: phagocytosis
GO:0061024: membrane organization
GO:0030316: osteoclast differentiation
-log10(P)
TAM_CCL4
WP3888: VEGFA-VEGFR2 signaling pathway
GO:0045785: positive regulation of cell adhesion
R-HSA-1280215: Cytokine Signaling in Immune system
GO:0051345: positive regulation of hydrolase activity
GO:0040017: positive regulation of locomotion
GO:0001775: cell activation
hsa05163: Human cytomegalovirus infection
GO:0050778: positive regulation of immune response
R-HSA-109582: Hemostasis
R-HSA-9012999: RHO GTPase cycle
GO:0042327: positive regulation of phosphorylation
R-HSA-9006934: Signaling by Receptor Tyrosine Kinases
hsa04666: Fc gamma R-mediated phagocytosis
hsa04933: AGE-RAGE signaling pathway in diabetic complications
GO:0010720: positive regulation of cell development
GO:0010810: regulation of cell-substrate adhesion
GO:0032103: positive regulation of response to external stimulus
WP4496: Signal transduction through IL1R
GO:0071363: cellular response to growth factor stimulus
GO:0001819: positive regulation of cytokine production
-log10(P)
C
Metabolic Pathway
Vitamin D calciferol metabolism
Vitamin C ascorbate metabolism
Vitamin B5 pantothenate metabolism
Vitamin B2 riboflavin metabolism
Vitamin B1 thiamin metabolism
Triglyceride metabolism
TP53 regulates metabolic genes
Surfactant metabolism
Sulfur amino acid metabolism
Sphingolipid metabolism
Sialic acid metabolism
Selenoamino acid metabolism
Regulation of lipid metabolism by pparalpha
Regulation of glycolysis by fructose 2 6 bisphosphate metabolism
Pyruvate metabolism and citric acid TCA cycle
Pyruvate metabolism
Pp2a mediated dephosphorylation of key metabolic factors
PI metabolism
Phospholipid metabolism
Phenylalanine metabolism
Phenylalanine and tyrosine metabolism
Peroxisomal lipid metabolism
Peptide hormone metabolism
Nicotinate metabolism
Metabolism of water soluble vitamins and cofactors
Metabolism of vitamins and cofactors
Metabolism of steroids
Metabolism of steroid hormones
Metabolism of RNA
Metabolism of porphyrins
Metabolism of polyamines
Metabolism of nucleotides
Metabolism of nitric oxide NOS3 activation and regulation
Metabolism of lipids
Metabolism of ingested semet sec mesec into H2SE
Metabolism of folate and pterines
Metabolism of fat soluble vitamins
Metabolism of cofactors
Metabolism of carbohydrates
Metabolism of angiotensinogen to angiotensins
Metabolism of amino acids and derivatives
Metabolism of amine derived hormones
Metabolic disorders of biological oxidation enzymes
Linoleic acid LA metabolism
Ketone body metabolism
Keratan sulfate keratin metabolism
Integration of energy metabolism
Inositol phosphate metabolism
Hyaluronan metabolism
Heparan sulfate heparin hs gag metabolism
Glyoxylate metabolism and glycine degradation
Glycosphingolipid metabolism
Glycosaminoglycan metabolism
Glycogen metabolism
Glutamate and glutamine metabolism
Glucose metabolism
Glucagon signaling in metabolic regulation
Fructose metabolism
Foxo mediated transcription of oxidative stress metabolic and neuronal genes
Fatty acid metabolism
Diseases of metabolism
Diseases of carbohydrate metabolism
Diseases associated with glycosaminoglycan metabolism
Defects in vitamin and cofactor metabolism
Defects in cobalamin B12 metabolism
Defects in biotin btn metabolism
Creatine metabolism
Cobalamin CBL vitamin B12 transport and metabolism
Class C3 metabotropic glutamate pheromone receptors
Chrebp activates metabolic gene expression
Chondroitin sulfate dermatan sulfate metabolism
Carnitine metabolism
Biotin transport and metabolism
Bile acid and bile salt metabolism
Aspartate and asparagine metabolism
Arachidonic acid metabolism
Alpha linolenic OMEGA3 and linoleic OMEGA6 acid metabolism
Abacavir transport and metabolism
BAM
cDC1
cDC2
MG
Mo
pDC
TAM_CCL4
TAM_GPNMB
TAM_RORA
TAM_SORL1
Value
1.00
0.75
0.50
0.25
0.00
Value
0.00
0.25
0.50
0.75
1.00
D
cDC1
pDC
BAM
TAM_GPNMB
TAM_RORA
cDC2
Mo
MG
TAM_CCL4
TAM_SORL1
High
Low
E
TAM_SORL1
TAM_GPNMB
TAM_RORA
TAM_CCL4
M2 Signature
M1 Signature
F
IRF8 Signature
cDC1
cDC2
pDC
TAM_CCL4
TAM_SORL1
TAM_GPNMB
TAM_RORA

Figure S2. Characterization of myeloid cells in ACP. Related to Figure 3.

(A) Heatmap showing the expression of functional genes in selected subclusters. The indicated functions are labelled on the left, while names of genes are on the right. **(B)** Bar plots of DEGs enriched pathways for TAM_SORL1, TAM_GPNMB and TAM_CCL4 using Metascape. P values are calculated based on the cumulative hypergeometric distribution. **(C)** Dot plot showing the metabolic activity analysis of myeloid subclusters coloured by scaled metabolic scores. **(D)** Heatmap showing the combined correlation between the inferred cell types across myeloid subclusters. **(E)** Scatterplots of M1 and M2 signature scores for each nucleus in 4 TAM subclusters. **(F)** Violin plot showing the IRF8 signature score in selected myeloid subclusters. The dashed lines indicate the median of the signature score, and the rhombus point in each violin represents its own median score.

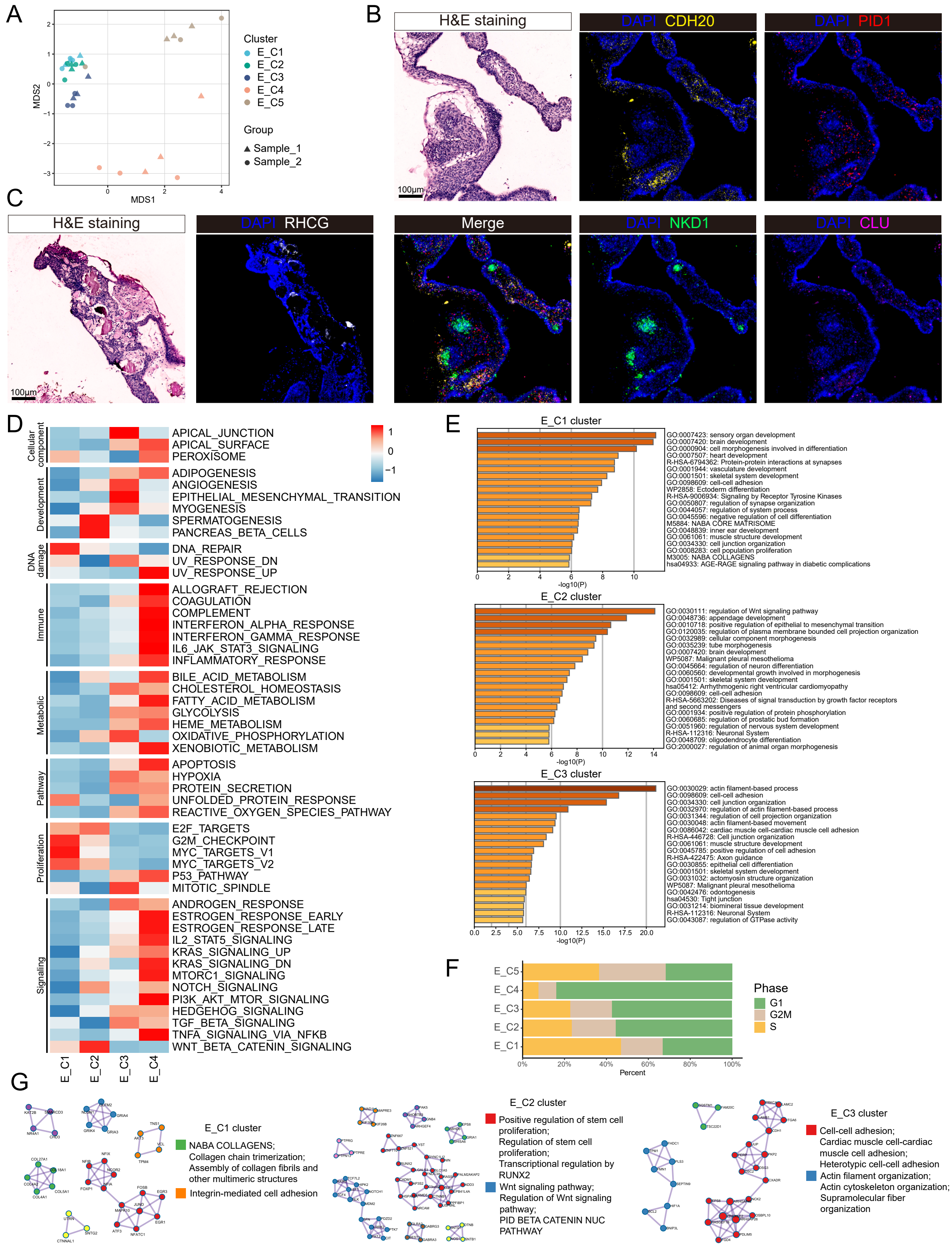
A
B
C
D
E
F
G
Cluster
E_C1
E_C2
E_C3
E_C4
E_C5
Group
Sample_1
Sample_2
MDS1
MDS2
H&E staining
DAPI CDH20
DAPI PID1
DAPI RHCG
Merge
DAPI NKD1
DAPI CLU
100μm
Cellular component
Development
DNA damage
Immune
Metabolic
Pathway
Proliferation
Signaling
APICAL_JUNCTION
APICAL_SURFACE
PEROXISOME
ADIPOGENESIS
ANGIOGENESIS
EPITHELIAL_MESENCHYMAL_TRANSITION
MYOGENESIS
SPERMATOGENESIS
PANCREAS_BETA_CELLS
DNA_REPAIR
UV_RESPONSE_DN
UV_RESPONSE_UP
ALLOGRAFT_REJECTION
COAGULATION
COMPLEMENT
INTERFERON_ALPHA_RESPONSE
INTERFERON_GAMMA_RESPONSE
IL6_JAK_STAT3_SIGNALING
INFLAMMATORY_RESPONSE
BILE_ACID_METABOLISM
CHOLESTEROL_HOMEOSTASIS
FATTY_ACID_METABOLISM
GLYCOLYSIS
HEME_METABOLISM
OXIDATIVE_PHOSPHORYLATION
XENOBIOTIC_METABOLISM
APOPTOSIS
HYPOXIA
PROTEIN_SECRETION
UNFOLDED_PROTEIN_RESPONSE
REACTIVE_OXYGEN_SPECIES_PATHWAY
E2F_TARGETS
G2M_CHECKPOINT
MYC_TARGETS_V1
MYC_TARGETS_V2
P53_PATHWAY
MITOTIC_SPINDLE
ANDROGEN_RESPONSE
ESTROGEN_RESPONSE_EARLY
ESTROGEN_RESPONSE_LATE
IL2_STAT5_SIGNALING
KRAS_SIGNALING_UP
KRAS_SIGNALING_DN
MTORC1_SIGNALING
NOTCH_SIGNALING
PI3K_AKT_MTOR_SIGNALING
HEDGEHOG_SIGNALING
TGF_BETA_SIGNALING
TNFA_SIGNALING_VIA_NFKB
WNT_BETA_CATENIN_SIGNALING
E_C1
E_C2
E_C3
E_C4
E_C1 cluster
GO:0007423: sensory organ development
GO:0007420: brain development
GO:0000904: cell morphogenesis involved in differentiation
GO:0007507: heart development
R-HSA-6794362: Protein-protein interactions at synapses
GO:0001944: vasculature development
GO:0001501: skeletal system development
GO:0098609: cell-cell adhesion
WP2858: Ectoderm differentiation
R-HSA-9006934: Signaling by Receptor Tyrosine Kinases
GO:0050807: regulation of synapse organization
GO:0044057: regulation of system process
GO:0045596: negative regulation of cell differentiation
M5884: NABA CORE MATRISOME
GO:0048839: inner ear development
GO:0061061: muscle structure development
GO:0034330: cell junction organization
GO:0008283: cell population proliferation
M3005: NABA COLLAGENS
hsa04933: AGE-RAGE signaling pathway in diabetic complications
-log10(P)
E_C2 cluster
GO:0030111: regulation of Wnt signaling pathway
GO:0048736: appendage development
GO:0010718: positive regulation of epithelial to mesenchymal transition
GO:0120035: regulation of plasma membrane bounded cell projection organization
GO:0032989: cellular component morphogenesis
GO:0035239: tube morphogenesis
GO:0007420: brain development
WP5087: Malignant pleural mesothelioma
GO:0045664: regulation of neuron differentiation
GO:0060560: developmental growth involved in morphogenesis
GO:0001501: skeletal system development
hsa05412: Arrhythmogenic right ventricular cardiomyopathy
GO:0098609: cell-cell adhesion
R-HSA-5663202: Diseases of signal transduction by growth factor receptors and second messengers
GO:0001934: positive regulation of protein phosphorylation
GO:0060685: regulation of prostatic bud formation
GO:0051960: regulation of nervous system development
R-HSA-112316: Neuronal System
GO:0048709: oligodendrocyte differentiation
GO:2000027: regulation of animal organ morphogenesis
E_C3 cluster
GO:0030029: actin filament-based process
GO:0098609: cell-cell adhesion
GO:0034330: cell junction organization
GO:0032970: regulation of actin filament-based process
GO:0031344: regulation of cell projection organization
GO:0030048: actin filament-based movement
GO:0086042: cardiac muscle cell-cardiac muscle cell adhesion
R-HSA-446728: Cell junction organization
GO:0061061: muscle structure development
GO:0045785: positive regulation of cell adhesion
R-HSA-422475: Axon guidance
GO:0030855: epithelial cell differentiation
GO:0001501: skeletal system development
GO:0031032: actomyosin structure organization
WP5087: Malignant pleural mesothelioma
GO:0042476: odontogenesis
hsa04530: Tight junction
GO:0031214: biomineral tissue development
R-HSA-112316: Neuronal System
GO:0043087: regulation of GTPase activity
Phase
G1
G2M
S
Percent
E_C1 cluster
NABA COLLAGENS; Collagen chain trimerization; Assembly of collagen fibrils and other multimeric structures
Integrin-mediated cell adhesion
E_C2 cluster
Positive regulation of stem cell proliferation; Regulation of stem cell proliferation; Transcriptional regulation by RUNX2
Wnt signaling pathway; Regulation of Wnt signaling pathway; PID BETA CATENIN NUC PATHWAY
E_C3 cluster
Cell-cell adhesion; Cardiac muscle cell-cardiac muscle cell adhesion; Heterotypic cell-cell adhesion
Actin filament organization; Actin cytoskeleton organization; Supramolecular fiber organization

Figure S3. Characterization of epithelial cells in ACP. Related to Figure 4.

(A) Pseudobulk-level Multidimensional Scaling (MDS) plot showing the overall transcriptional similarity of 5 epithelial cell subclusters found in all samples. **(B-C)** asmFISH of ACP tissues, showing the specific markers of epithelial subtypes. CDH20 (yellow) for E_C1, NKD1 (green) for E_C2, PID1 (red) for E_C3, RHCG (white) for E_C4 and CLU (purple) for E_C5 are shown in individual channels respectively. H&E-stained adjacent section to show histology. Scale bar: 100 μm. **(D)** Heatmap showing the activation of 50 hallmark gene sets scored by GSVA among epithelial subclusters, except the E_C5 cluster. All scores are normalized. The process category is labelled on the left. **(E)** Bar plots of DEG-enriched pathways for selected epithelial subclusters using Metascape. Names of subclusters are labelled on the top. **(F)** Stacked bar plot showing the proportion of the cell cycle phase in each epithelial subcluster. Colours match the phases. **(G)** The PPI network showing the complexes automatically identified by the MCODE algorithm in Metascape, coloured by each identity. Two major functional labels are listed on the right side.

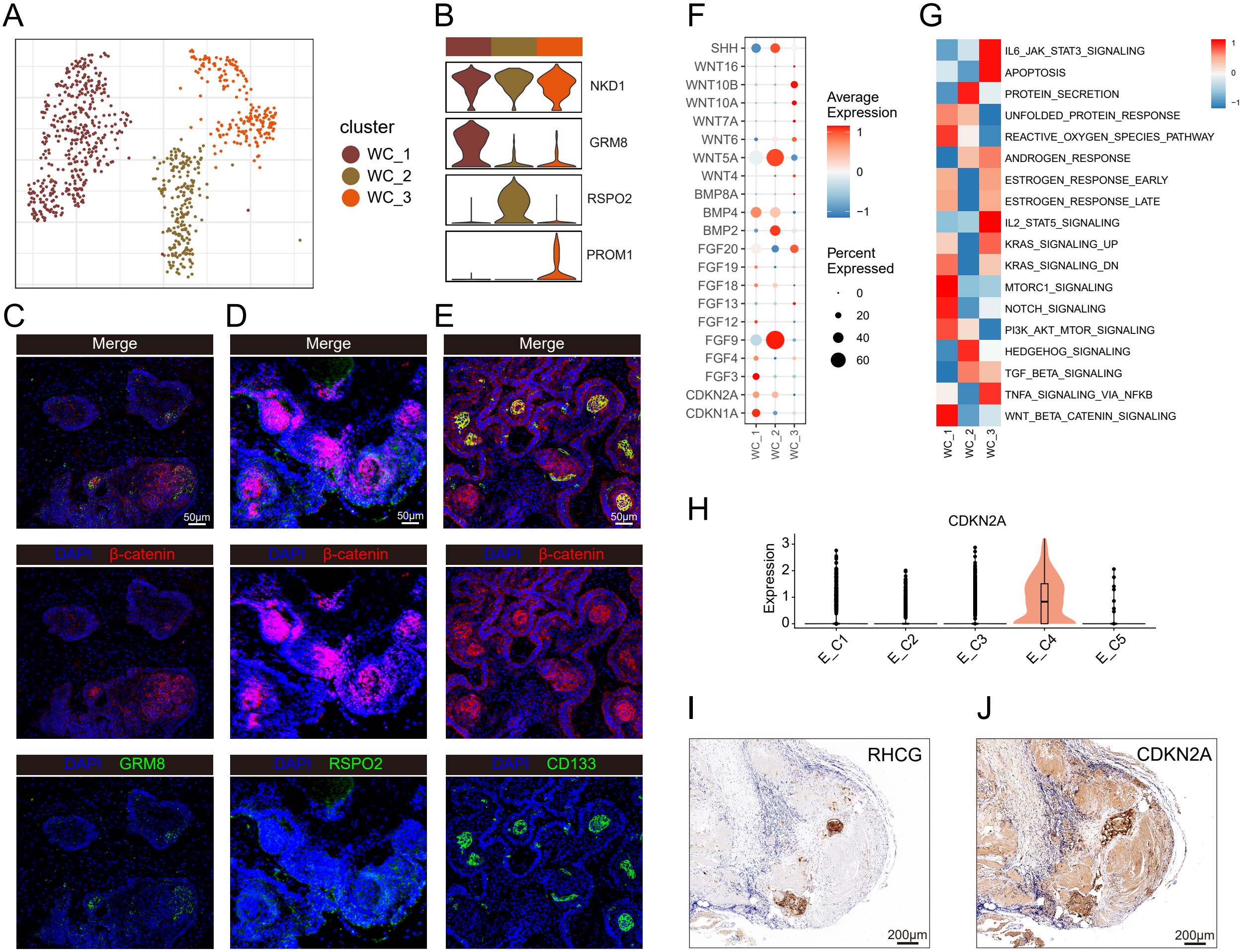
A
B
C
D
E
F
G
H
I
J
cluster
WC_1
WC_2
WC_3
NKD1
GRM8
RSPO2
PROM1
Merge
50μm
DAPI β-catenin
DAPI GRM8
DAPI RSPO2
DAPI CD133
SHH
WNT16
WNT10B
WNT10A
WNT7A
WNT6
WNT5A
WNT4
BMP8A
BMP4
BMP2
FGF20
FGF19
FGF18
FGF13
FGF12
FGF9
FGF4
FGF3
CDKN2A
CDKN1A
Average Expression
1
0
−1
Percent Expressed
0
20
40
60
IL6_JAK_STAT3_SIGNALING
APOPTOSIS
PROTEIN_SECRETION
UNFOLDED_PROTEIN_RESPONSE
REACTIVE_OXYGEN_SPECIES_PATHWAY
ANDROGEN_RESPONSE
ESTROGEN_RESPONSE_EARLY
ESTROGEN_RESPONSE_LATE
IL2_STAT5_SIGNALING
KRAS_SIGNALING_UP
KRAS_SIGNALING_DN
MTORC1_SIGNALING
NOTCH_SIGNALING
PI3K_AKT_MTOR_SIGNALING
HEDGEHOG_SIGNALING
TGF_BETA_SIGNALING
TNFA_SIGNALING_VIA_NFKB
WNT_BETA_CATENIN_SIGNALING
CDKN2A
Expression
0
1
2
3
E_C1
E_C2
E_C3
E_C4
E_C5
RHCG
CDKN2A
200μm

Figure S4. Characterization of epithelial subtypes in ACP. Related to Figure 4.

(A) UMAP of E_C2 re-clustered into 3 subclusters in different colours. **(B)** Violin plot showing the expression of marker genes. **(C)** Representative staining of WC_1. β-catenin (red) and GRM8 (green) are shown in individual channels with DAPI (blue). Scale bar: 50 μm. **(D)** Representative FISH of WC_2. β-catenin (red) and RSPO2 (green) are shown in individual channels with DAPI (blue). Scale bar: 50 μm. **(E)** Representative staining of WC_3. β-catenin (red) and CD133 (green) which encoded by PROM1 are shown in individual channels with DAPI (blue). Scale bar: 50 μm. **(F)** Dot plot showing signal factors for 3 WC clusters coloured by expression levels. **(G)** Heatmap showing the activation of hallmark gene sets scored by GSVA among E_C2 subclusters. All scores are normalized. **(H)** Violin plot showing the expression of selected genes (CDKN2A) in epithelial cell subclusters. **(I-J)** IHC showing positive staining of RHCG and CDKN2A in similar location. Scale bar: 200 μm.

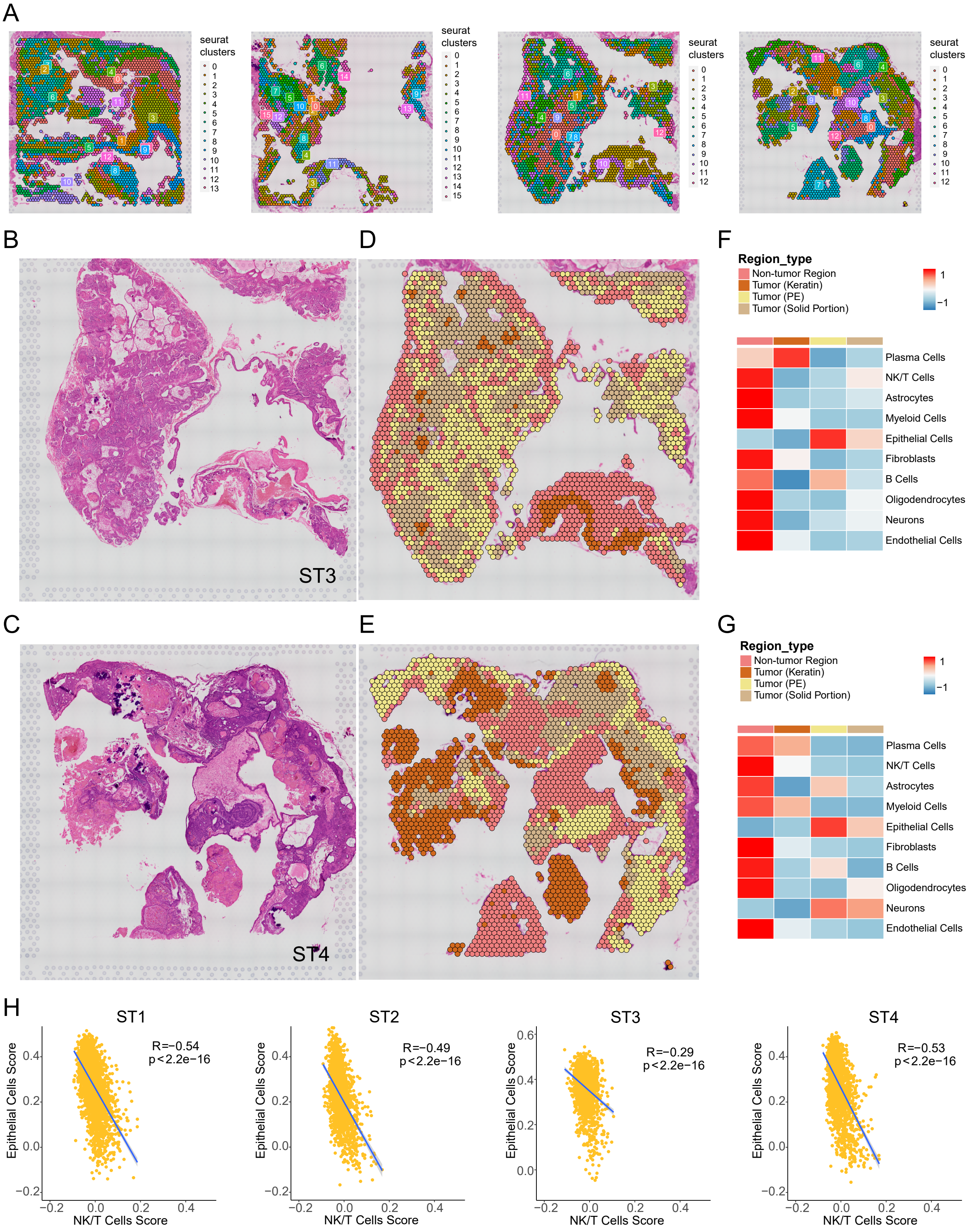
A
seurat clusters
B
ST3
D
F
Region_type
Non-tumor Region
Tumor (Keratin)
Tumor (PE)
Tumor (Solid Portion)
Plasma Cells
NK/T Cells
Astrocytes
Myeloid Cells
Epithelial Cells
Fibroblasts
B Cells
Oligodendrocytes
Neurons
Endothelial Cells
C
ST4
E
G
Region_type
Non-tumor Region
Tumor (Keratin)
Tumor (PE)
Tumor (Solid Portion)
Plasma Cells
NK/T Cells
Astrocytes
Myeloid Cells
Epithelial Cells
Fibroblasts
B Cells
Oligodendrocytes
Neurons
Endothelial Cells
H
ST1
R=−0.54
p<2.2e−16
ST2
R=−0.49
p<2.2e−16
ST3
R=−0.29
p<2.2e−16
ST4
R=−0.53
p<2.2e−16
Epithelial Cells Score
NK/T Cells Score

Figure S5. Spatial features of 10 major cell types in ACP. Related to Figure 5.

(A) Spatial sections showing all small clusters in each ST sample before integration. The sections, from left to right, are from ST1, ST2, ST3 and ST4 samples. **(B-C)** H&E staining images of sections showing the distinct pathological features of ST3 and ST4 samples. **(D-E)** Spatial spots are separated into different regions with distinct pathological features through unbiased clustering in ST3 and ST4 samples. **(F-G)** Heatmaps showing the mean signature scores of 10 major cell types in each divided region. The signature genes of 10 major cell types are based on our snRNA-seq data. **(H)** Correlations of the signature scores of NK/T cells and epithelial cells for each spatial spot in the ST1 to ST4 samples. The error band shows the 95% confidence interval.

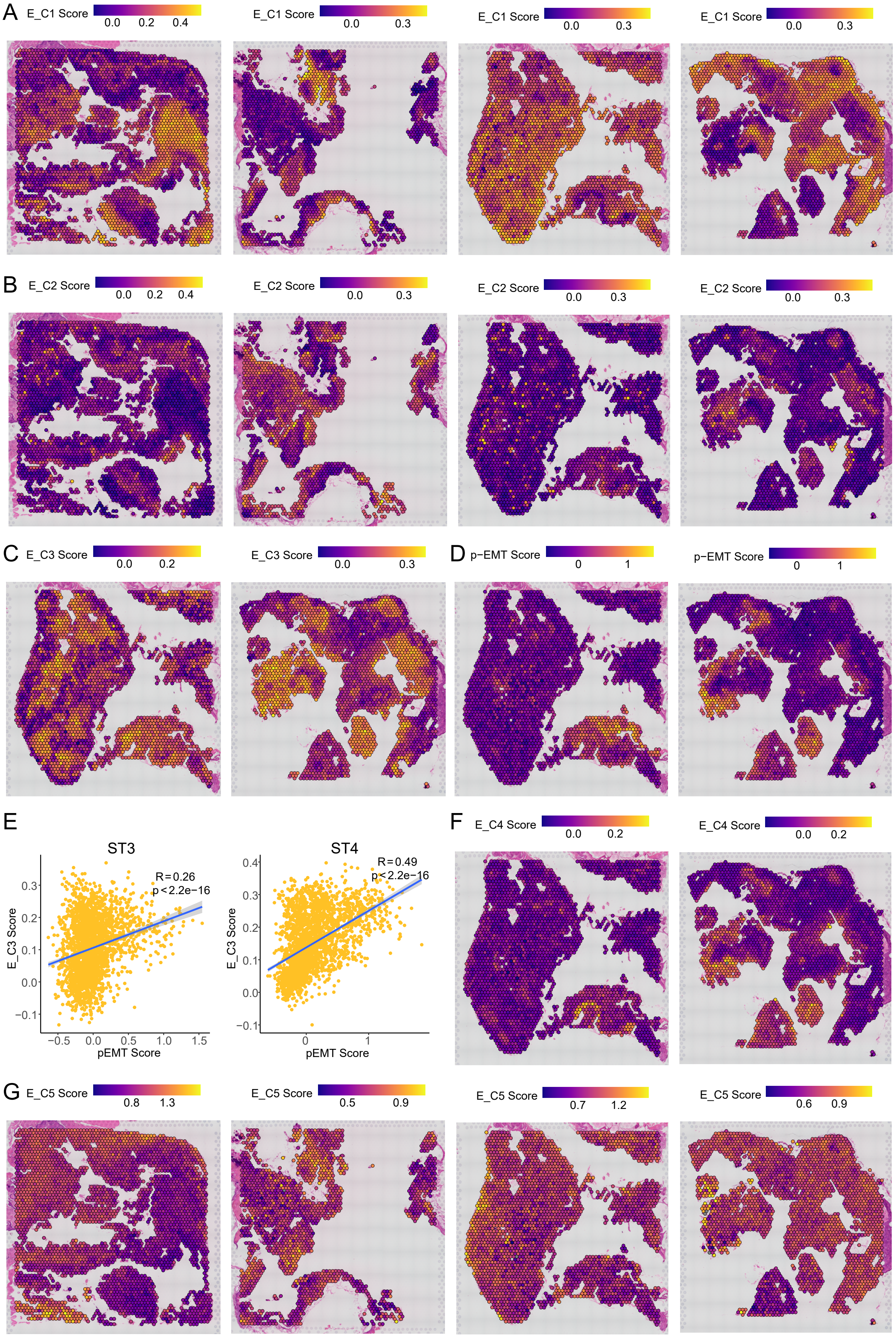
A
E_C1 Score 0.0 0.2 0.4
E_C1 Score 0.0 0.3
E_C1 Score 0.0 0.3
E_C1 Score 0.0 0.3
B
E_C2 Score 0.0 0.2 0.4
E_C2 Score 0.0 0.3
E_C2 Score 0.0 0.3
E_C2 Score 0.0 0.3
C
E_C3 Score 0.0 0.2
E_C3 Score 0.0 0.3
D
p−EMT Score 0 1
p−EMT Score 0 1
E
ST3
R=0.26
p<2.2e−16
E_C3 Score
pEMT Score
ST4
R=0.49
p<2.2e−16
E_C3 Score
pEMT Score
F
E_C4 Score 0.0 0.2
E_C4 Score 0.0 0.2
G
E_C5 Score 0.8 1.3
E_C5 Score 0.5 0.9
E_C5 Score 0.7 1.2
E_C5 Score 0.6 0.9

Figure S6. Spatial visualization of different subclusters. Related to Figure 5.

(A) Signature scores of the E_C1 cluster in each spot on ST sections, from left to right, named ST1 to ST4 samples. **(B)** Signature scores of the E_C2 cluster in each spot on ST sections, from left to right, named ST1 to ST4 samples. **(C)** Signature scores of the E_C3 cluster in each spot on ST sections from ST3 and ST4 samples. **(D)** Scores of the pEMT signature in each spot on ST sections from ST3 and ST4 samples. **(E)** The correlations of the signature score of pEMT and the E_C3 cluster for each spatial spot in the ST3 and ST4 samples. The error band shows the 95% confidence interval. **(F)** Signature scores of the E_C4 cluster in each spot on ST sections from ST3 and ST4 samples. **(G)** Signature scores of the E_C5 cluster in each spot on ST sections, from left to right, named ST1 to ST4 samples.

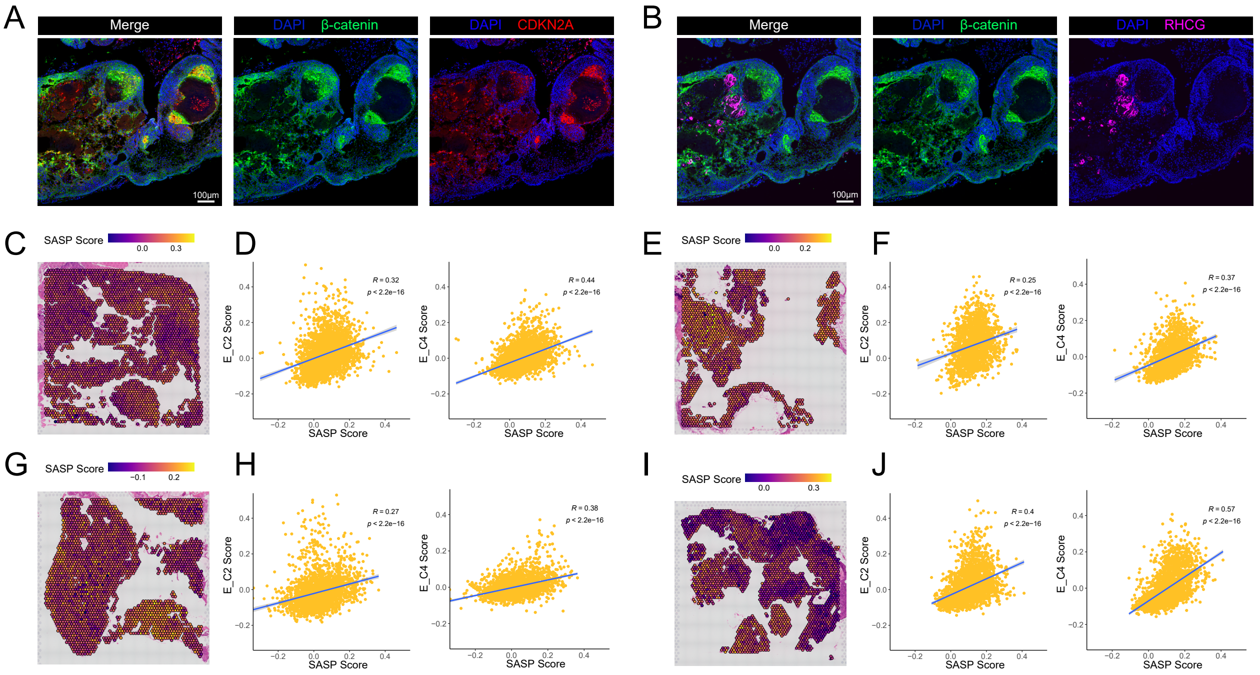
A
Merge
DAPI β-catenin
DAPI CDKN2A
100μm
B
Merge
DAPI β-catenin
DAPI RHCG
100μm
C
SASP Score
0.0
0.3
D
R = 0.32
p < 2.2e−16
E_C2 Score
SASP Score
R = 0.44
p < 2.2e−16
E_C4 Score
SASP Score
E
SASP Score
0.0
0.2
F
R = 0.25
p < 2.2e−16
E_C2 Score
SASP Score
R = 0.37
p < 2.2e−16
E_C4 Score
SASP Score
G
SASP Score
−0.1
0.2
H
R = 0.27
p < 2.2e−16
E_C2 Score
SASP Score
R = 0.38
p < 2.2e−16
E_C4 Score
SASP Score
I
SASP Score
0.0
0.3
J
R = 0.4
p < 2.2e−16
E_C2 Score
SASP Score
R = 0.57
p < 2.2e−16
E_C4 Score
SASP Score

Figure S7. Spatial feature of SASP related to E_C2 and E_C4. Related to Figure 5.

(A) Representative staining of areas with WC and RHCG^{+} epithelial cells. β-catenin (green) and CDKN2A (red) are shown in individual channels with DAPI (blue). Scale bar: 100 μm. **(B)** Staining of adjacent section. β-catenin (green) and RHCG (purple) are shown in individual channels with DAPI (blue). Scale bar: 100 μm. **(C)** Signature score of SASP in ST1 section. **(D)** The correlations of the signature score of SASP and E_C2/E_C4 clusters for each spatial spot in ST1 section. The error band shows the 95% confidence interval. **(E)** Signature score of SASP in ST2 section. **(F)** The correlations of the signature score of SASP and E_C2/E_C4 clusters for each spatial spot in ST2 section. The error band shows the 95% confidence interval. **(G)** Signature score of SASP in ST3 section. **(H)** The correlations of the signature score of SASP and E_C2/E_C4 clusters for each spatial spot in ST3 section. The error band shows the 95% confidence interval. **(I)** Signature score of SASP in ST4 section. **(J)** The correlations of the signature score of SASP and E_C2/E_C4 clusters for each spatial spot in ST4 section. The error band shows the 95% confidence interval.

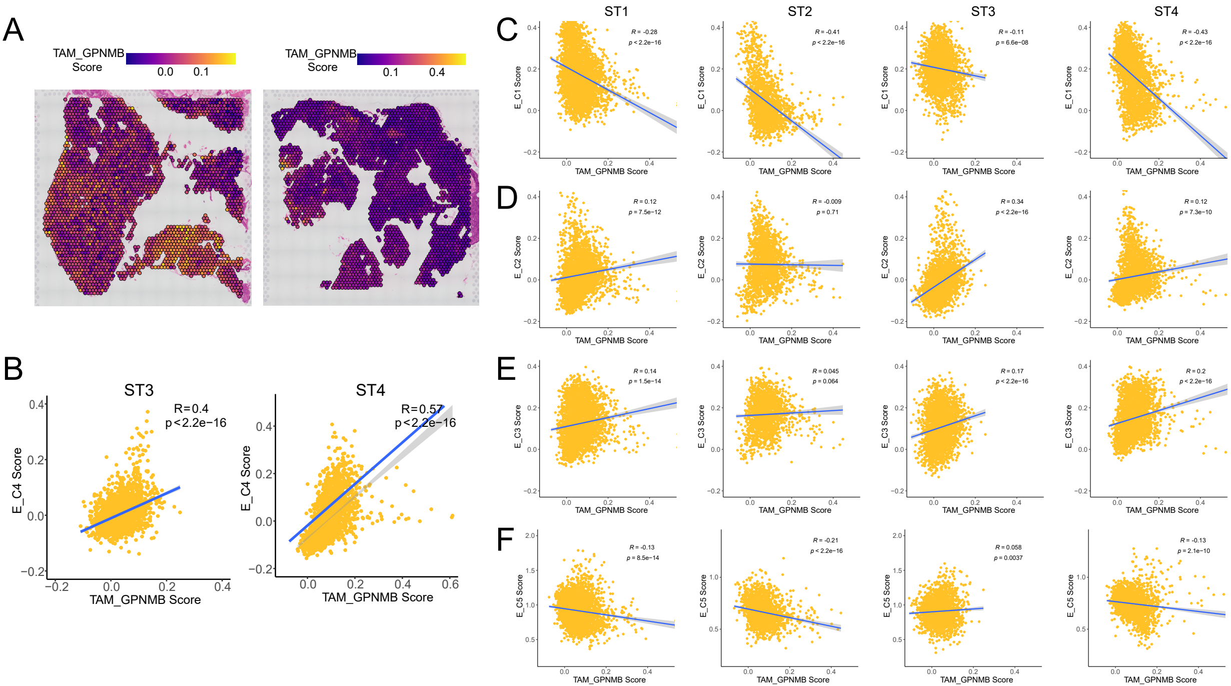

A
TAM_GPNMB Score
0.0
0.1
TAM_GPNMB Score
0.1
0.4
B
ST3
R=0.4
p<2.2e−16
E_C4 Score
TAM_GPNMB Score
ST4
R=0.57
p<2.2e−16
E_C4 Score
TAM_GPNMB Score
C
ST1
ST2
ST3
ST4
E_C1 Score
TAM_GPNMB Score
R = -0.28
p < 2.2e−16
R = -0.41
p < 2.2e−16
R = -0.11
p = 6.6e−08
R = -0.43
p < 2.2e−16
D
E_C2 Score
TAM_GPNMB Score
R = 0.12
p = 7.5e−12
R = -0.009
p = 0.71
R = 0.34
p < 2.2e−16
R = 0.12
p = 7.3e−10
E
E_C3 Score
TAM_GPNMB Score
R = 0.14
p = 1.5e−14
R = 0.045
p = 0.064
R = 0.17
p < 2.2e−16
R = 0.2
p < 2.2e−16
F
E_C5 Score
TAM_GPNMB Score
R = -0.13
p = 8.5e−14
R = -0.21
p < 2.2e−16
R = 0.058
p = 0.0037
R = -0.13
p = 2.1e−10

Figure S8. Spatial correlation of TAM_GPNMB and epithelial subclusters. Related to Figure 5.

(A) Signature scores of TAM_GPNMB in each spot on ST sections from ST3 and ST4 samples. **(B)** The correlations of the signature scores of the TAM_GPNMB and E_C4 clusters for each spatial spot in the ST3 and ST4 samples, respectively. The error band indicates the 95% confidence interval. **(C)** The correlations of the signature scores of the TAM_GPNMB and E_C1 clusters for each spatial spot in all ST sections, respectively. The error band indicates the 95% confidence interval. **(D)** The correlations of the signature scores of the TAM_GPNMB and E_C2 clusters for each spatial spot in all ST sections, respectively. The error band indicates the 95% confidence interval. **(E)** The correlations of the signature scores of the TAM_GPNMB and E_C3 clusters for each spatial spot in all ST sections, respectively. The error band indicates the 95% confidence interval. **(F)** The correlations of the signature scores of the TAM_GPNMB and E_C5 clusters for each spatial spot in all ST sections, respectively. The error band indicates the 95% confidence interval.

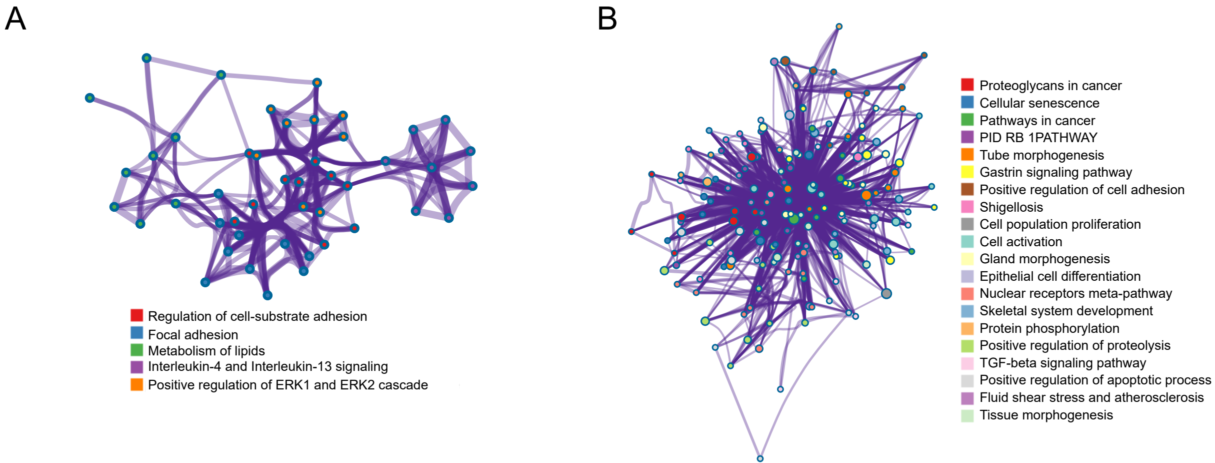
A
Regulation of cell-substrate adhesion
Focal adhesion
Metabolism of lipids
Interleukin-4 and Interleukin-13 signaling
Positive regulation of ERK1 and ERK2 cascade
B
Proteoglycans in cancer
Cellular senescence
Pathways in cancer
PID RB 1PATHWAY
Tube morphogenesis
Gastrin signaling pathway
Positive regulation of cell adhesion
Shigellosis
Cell population proliferation
Cell activation
Gland morphogenesis
Epithelial cell differentiation
Nuclear receptors meta-pathway
Skeletal system development
Protein phosphorylation
Positive regulation of proteolysis
TGF-beta signaling pathway
Positive regulation of apoptotic process
Fluid shear stress and atherosclerosis
Tissue morphogenesis

Figure S9. Enrichment analysis of predicted target genes. Related to Fig. 7.

(A) For predicted target genes in TAM_GPNMB, enrichment analysis in Metascape was used to identify enriched pathways and processes, and the network plot shows the subset of enriched terms coloured by term cluster. **(B)** For predicted target genes in both E_C3 and E_C4 clusters, enrichment analysis in Metascape was used to identify enriched pathways and processes.
